# The Interplay Between Quality of Life and Resilience Factors in Later Life: A Network Analysis

**DOI:** 10.3389/fpsyg.2021.752564

**Published:** 2021-11-15

**Authors:** Lotte P. Brinkhof, Karoline B. S. Huth, Jaap M. J. Murre, Sanne de Wit, Harm J. Krugers, K. Richard Ridderinkhof

**Affiliations:** ^1^Department of Psychology, Faculty of Behavioural and Social Sciences, University of Amsterdam, Amsterdam, Netherlands; ^2^Centre for Urban Mental Health, University of Amsterdam, Amsterdam, Netherlands; ^3^Amsterdam Brain & Cognition (ABC), University of Amsterdam, Amsterdam, Netherlands; ^4^Department of Psychiatry, Amsterdam University Medical Centre, Amsterdam, Netherlands; ^5^Faculty of Science, Swammerdam Institute for Life Sciences, University of Amsterdam, Amsterdam, Netherlands

**Keywords:** quality of life, resilience, older adults, network analysis, self-management ability, coping, positive appraisal, physical activity

## Abstract

Age-related challenges and transitions can have considerable social, psychological, and physical consequences that may lead to significant changes in quality of life (QoL). As such, maintaining high levels of QoL in later life may crucially depend on the ability to demonstrate resilience (i.e., successful adaptation to late-life challenges). The current study set out to explore the interplay between several resilience factors, and how these contribute to the realization and maintenance of (different facets of) QoL. Based on the previous work, we identified behavioral coping, positive appraisal, self-management ability, and physical activity as key resilience factors. Their interplay with (various facets of) QoL, as measured with the WHOQOL-OLD, was established through network analysis. In a sample of community-dwelling older adults (55+; *N* = 1,392), we found that QoL was most strongly (and directly) related to positive appraisal style and self-management ability. Among those, self-efficacy seemed to be crucial. It connected directly to “satisfaction with past, present, and future activities,” a key facet of QoL with strong interconnections to other QoL facets. Our analysis also identified resilience factor(s) with the potential to promote QoL when targeted by training, intervention, or other experimental manipulation. The appropriate set of resilience factors to manipulate may depend on the goal and/or facet of QoL that one aims to improve.

## Introduction

Aging gives rise to certain challenges and transitions that are often unavoidable (e.g., cognitive decline, physical deterioration, or loss of spouse and friends; Janssen et al., 2002; Newman et al., 2003; Glisky, 2007; Vasconcelos et al., 2016; Wang et al., 2020). These can have considerable social, psychological, or physical consequences (Fried et al., 2015; Valtorta et al., 2016; Jaul and Barron, 2017; Ohrnberger et al., 2017a,b). While some older adults will be able to compensate for these changes, others may lack the capacity to adequately self-regulate and manage them, increasing the risk to experience declines in *quality of life* (QoL; e.g., Hildon et al., 2010; Hayman et al., 2017). This study set out to explore how positive resources, or *resilience factors*, can contribute to the realization and maintenance of QoL in later life and thereby promote *successful aging*. A secondary aim was to identify those resilience factor(s) that hold potential to promote QoL when targeted by trainings, interventions, or other kinds of experimental manipulations, thereby gaining insight into how older adults’ control of their own lives (or *empowerment*) could be enhanced.

While successful aging has commonly been defined in terms of physical health and functioning, we consider it as the process of developing and maintaining the functional ability and competence that enables well-being and QoL at old age (cf. World Health Organization’s definition of healthy aging; WHO, 2020a). This suggests that the key to thriving in late life may be the ability to demonstrate resilience to age-related challenges and transitions (Greve and Staudinger, 2006; Harris, 2008; Netuveli and Blane, 2008; Fry and Keyes, 2010; MacLeod et al., 2016). Resilience can be defined as the process of successfully adapting to difficult or challenging life experiences, especially through mental, emotional, and behavioral flexibility and adjustment to external and internal demands (American Psychological Association, 2020). In late life, resilience can be conceptualized as a protective characteristic that promotes the maintenance of functional ability or competence, well-being, and QoL despite the presence of internal or external age-related threats, risks, or adversity (Ryff et al., 1998; Mehta et al., 2008; Hildon et al., 2010; Stephens et al., 2015; Hayman et al., 2017). While resilience has often been measured as a personality characteristic or trait (Windle et al., 2011), it has increasingly been evaluated in terms of potential health outcomes as well (e.g., QoL or well-being; Netuveli et al., 2008; Hildon et al., 2010; Chmitorz et al., 2018; Kalisch et al., 2020), characterized by multiple *resilience factors*. In the current study, we build on the latter outcome-based perspective by focusing on the *interplay* between such factors in relation to QoL specifically (see also Hildon et al., 2008, 2010). This approach acknowledges the complex and emergent nature of resilience (Brinkhof et al., 2021).

According to the World Health Organization, QoL can be defined as “*individuals’ perceptions of their position in life in the context of the culture and value systems in which they live and in relation to their goals, expectations, standards and concerns*” (WHO, 1997). It refers to the cognitive appraisal of one’s life, rather than the emotional response to it (which comprises the concept of well-being), and therefore extends beyond the mere assessment of health status and functioning (Upton and Upton, 2015). QoL is often used as an umbrella term in aging research to describe a number of outcomes that are believed to be important in the lives of older adults (Rejeski and Mihalko, 2001; Upton and Upton, 2015). Accordingly, it has been recognized as a pillar for pleasant living in later life (Ekerdt et al., 2017), and the maintenance of good QoL has been endorsed as an important focal point for governments and health policies (Bowling, 2007; WHO, 2020b). The most frequently used instrument to assess QoL in older adults is the World Health Organization Quality of Life-Older Adults Module (WHOQOL-OLD). This instrument recognizes the multidimensionality of QoL and comprises six facets that are considered especially important to older adults. These include sensory abilities (SAB); autonomy; satisfaction with past, present, and future activities and achievements in life; social participation (SOP); concerns, worries and fears about death and dying (DAD); and being able to have personal and intimate relationships.

Researchers have tried to ascertain the variety of resources that may help to shield individuals against challenges and setbacks or reduce their negative consequences (Clark et al., 2011) and have thus identified a wide range of resilience factors (e.g., Greve and Staudinger, 2006; Fontes and Neri, 2015; MacLeod et al., 2016). For the current study, we focused on a small selection of factors that appear particularly relevant in relation to QoL and its facets, and hold great potential for intervention. A factor that is widely studied is *coping ability*. Coping generally refers to the cognitive and behavioral efforts (e.g., emotion regulation and problem solving) implemented to modulate internal or external demands that are appraised as taxing or exceeding personal resources (Lazarus and Folkman, 1984; Endler and Parker, 1990; Garnefski et al., 2001). Adaptive coping skills are thought to be critical for adaptation at old age (Gamrowska and Steuden, 2014; MacLeod et al., 2016) and have been associated with higher QoL (Hildon et al., 2010; Rey and Extremera, 2016; León-Navarrete et al., 2017). Two recently developed instruments that capture various cognitive (emotion regulation) and behavioral coping strategies are the Positive Appraisal Style Scale (PASS) and the Behavioral Coping Scale (BCS). These scales were developed based on a factor analysis (see Kalisch et al., 2020) on the subscale level of the brief COPE (Carver, 1997), CERQ-short (Garnefski and Kraaij, 2006a), and two additional items on distanced stressor appraisal. The 14-item PASS reflects positive appraisal content and processes based on seven subscales (i.e., distanced stressor appraisal, positive reappraisal, acceptance, putting into perspective, refocus on planning, positive refocusing and humor). The 8-item BCS reflects less cognitive and more behavioral coping and includes four subscales: use of instrumental support, emotional support seeking, venting of emotions, and planning/acting out. It has been confirmed that both the PASS and BCS positively predict outcome-based resilience (i.e., General Health Questionnaire; see Kalisch et al., 2020; Veer et al., 2020). Moreover, some of the subcomponents of the scales have also been linked frequently to positive (mental) health outcomes (Garnefski et al., 2001; Kraaij et al., 2002; Garnefski and Kraaij, 2006b; Raut et al., 2014; Nowlan et al., 2015) and have been shown to be modifiable (Garland et al., 2009; Hanley and Garland, 2014; Satorres et al., 2018). Yet, the exact relationships with QoL have not been investigated.

Although studied less thoroughly, and not directly related to resilience in previous research, self-management ability (SMA) may also comprise an important factor that underpins resilience in old age (Schuurmans et al., 2004; Cramm et al., 2014). It has been suggested that successful aging, and the realization and maintenance of well-being in particular, depends on the proactive management of *external* resources (social and physical, e.g., friends and physical fitness; Steverink et al., 2005), that often decline with age. Within the context of the Self-Management of Well-being theory, Steverink et al. (2005) identified six SMAs that are important to adequately manage one’s life and external resources in the process of aging (Nieboer and Lindenberg, 2002; Steverink et al., 2005; Cramm et al., 2012a). These include the ability to take initiative; invest in resources for long-term benefits; be self-efficacious with regard to managing resources; keep a positive frame of mind regarding the future; maintain care of a variety of resources (providing a buffer in case a resource is lost); and maintain multifunctional resources (i.e., yielding various benefits at the same time: killing two birds with one stone). An example of a multifunctional resource is a friend/spouse, who can satisfy one’s need for affection, but also supports the fulfilment of other needs (e.g., sharing burdens or stimulation, by jointly participating in interesting activities). All facets were identified under the assumption that they should in principle be modifiable by training, interventions, or other kinds of manipulations (e.g., support of community nurses) to improve health outcomes, which has subsequently been confirmed in several studies (e.g., Schuurmans, 2004; Frieswijk et al., 2006; Kremers et al., 2006; Cramm and Nieboer, 2017). Unlike the fundamental and theoretical relationship with well-being, the role of self-management and how the orchestrated use of these abilities may help to sustain QoL in later life is relatively underexplored (e.g., Cramm and Nieboer, 2017).

In addition to these psychosocial factors, being physically active has also been identified as an important factor that could promote resilience (e.g., Childs and de Wit, 2014). Besides improving (physical) health (e.g., Arroll and Beaglehole, 1992; Vella et al., 2001; Taylor, 2014), physical activity has also been related to a reduced incidence of mental health problems (Goodwin, 2003). Moreover, physical activity interventions have been shown to improve mental well-being and QoL reports (Conn et al., 2009; Windle et al., 2010; Figueira et al., 2012). The practice of physical activity by older adults may also strengthen their abilities to manage or cope with challenges or difficulties (Ho et al., 2015; Ávila et al., 2018) and interact with factors such as coping, appraisal, and self-management, thereby indirectly promoting QoL. On the other hand, it has also been proposed that strengthening resilience may improve the adherence to exercise behaviors (e.g., Resnick and Inguito, 2011). Surprisingly, the number of studies investigating such relationships, especially among older adults, is scarce. More studies are needed to understand how exactly being physically active plays a role in building resilience at old age (Rejeski and Mihalko, 2001).

In the current study, we explored the interplay between behavioral coping (BC), positive appraisal style, SMA, and physical activity (PHY), and how these affect (the facets of) QoL. While Gerino et al. (2017) have also looked at the interplay between QoL and multiple psychosocial factors, including general self-efficacy and personality characteristics that are thought to enhance individual adaptation, we aim to build upon recent findings and explore the (relative) contribution of the aforementioned factors. Moreover, we will employ the WHOQOL-OLD, and the individual facets of that construct, which is more tailored to older adults than the WHOQOL-BREF used by Gerino et al. (2017).

In order to obtain a comprehensive view of these interactions, we performed several network analyses in a sample of healthy, community-dwelling older adults (*N*=1,392, 55years and above). This allowed us to visualize the interplay between (facets of) QoL and the aforementioned resilience factors. Of note, the aim of this study was not to characterize resilience in terms of stability of the network (e.g., weakly connected networks are considered more stable/resilient; see, e.g., Borsboom, 2017; Kalisch et al., 2019), but rather to focus on the interplay between QoL as an outcome of resilience and some factors that are thought to underpin resilience in later life. Specifically, we sought to determine the relative strengths of the contributions of the empirically supported resilience factors to overall QoL, as well as to the six individual facets. Given the novelty of using a complex system approach to elaborate on the interplay between resilience factors, and how they in turn affect QoL, we had no strong *a priori* hypotheses on the interrelations. Nevertheless, we expected that all factors would be positively related to (different facets of) QoL, either directly or indirectly. This allowed us to also assess which factors might hold potential for modulating QoL through intervention, by examining the patterns and strengths of the relationships, as well as their relative importance as predictors of QoL within the network. Unlike previous studies that have looked at various resilience factors in isolation from each other (see MacLeod et al., 2016), our approach can widen our understanding of how resilience in later life emerges out of the interactions between some of its underlying components. Unsuspected pathways (e.g., mediation effects) through which QoL in later life could potentially be realized and maintained may be revealed accordingly. Hence, the current study can provide unique insights on how certain characteristics can contribute to higher levels of resilience in later life.

## Materials and Methods

### Sample Characteristics

Data are derived from an ongoing large-scale online study on successful aging and resilience (Brinkhof et al., 2021; approved by the local ethics committee of the University of Amsterdam, 2020-DP-12556), where participants complete an inventory including several questionnaires and tests that cover a multitude of relevant factors from multiple domains (e.g., physical, psychological, cognitive, social, and environmental). It took approximately 1–1.5h to complete the entire inventory, but participation could be divided over several days. Participants were eligible to enroll in this online inventory when they were 55years or older, living in the Netherlands and had no Alzheimer’s Disease, vascular dementia, frontotemporal dementia, Lewy body dementia, or other dementia diagnosis. While (apparent) age-related declines may generally be expected at a somewhat older age, some individuals already experience gradual declines in physical or mental capacity at a younger age. Hence, a relatively broad age range was adopted. Other exclusion criteria were insufficient command of the Dutch language, impaired vision, or not being able to perform the operations required to successfully use a computer or laptop independently (i.e., mouse clicks, pressing keys on the keyboard). Participants were forced to answer each question in order to proceed through the inventory. The first 1,500 participants that completed the entire inventory within 14days after they started with the first part of the study were included in the current study. These participants took part in the study between October 5, 2020, and January 11, 2021, in the midst of the COVID-19 pandemic.

### Materials

All materials included in the inventory are reported and described in (Brinkhof et al., 2021). The variables included in the current study are briefly repeated here.

#### Quality of Life

The Dutch language version of the WHOQOL-OLD (Power et al., 2005; Gobbens and van Assen, 2016) was used as measure of QoL. The measure comprises 24 items divided into six subscales of four items each: (1) sensory abilities (SAB; e.g., “To what extent do impairments to your senses (e.g., hearing, vision, taste, smell, touch) affect your daily life?”), (2) autonomy (AUT; e.g., “How much freedom do you have to make your own decisions?”), (3) past, present and future activities (PPF; e.g., “How satisfied are you with what you have achieved in life?”), (4) SOP (e.g., “To what extent do you feel that you have enough to do each day?”), (5) DAD (e.g., “How scared are you of dying?”), and (6) intimacy (INT; e.g., “To what extent do you feel a sense of companionship in your life?”). Responses were scored on 5-point Likert scales, with different wording, and summed across each subscale, as well as to a total QoL score. Higher scores (per scale) indicated better QoL. All items corresponding to the fifth subscale and some of items of the first and second subscale were reverse scored prior to summation.

#### Positive Appraisal Style and Behavioral Coping

The PASS and BCS were used to measure PAS and BC, respectively. While the BCS only constitutes items from the brief COPE, the PASS includes subscales from both the brief COPE, CERQ short and the two self-generated items (on distanced stressor appraisal). For that reason, participants were asked to fill out the “shortened COPE,” including all subscales from the BCS and the humor subscale from the PASS, and the “shortened CERQ,” including all remaining six subscales of the PASS, of which one constitutes the two self-generated items. All 10 items of the shortened COPE (including, e.g., “I’ve been getting emotional support from others”) were scored on a 4-point Likert Scale (1=not at all, 2=a little bit, 3=quite a lot, 4=a lot) and 12-items of the shortened CERQ (e.g., “I think that I have to accept that this has happened”) were scored on a 5-point Likert scale [1=(almost) never, 2=sometimes, 3=regularly, 4=often, 5=(almost) always]. The items from the subscales of the BCS were summed to a total BCS score of 8–32. The PASS score was determined by taking the average of the z-normalized scores of the items derived from the shortened COPE, the CERQ, and the self-generated items.

#### Self-Management Ability

The Self-Management Ability Scale (SMAS)-18 was used to measure SMA (Cramm et al., 2012b). The 18-item version of the original 30-item SMAS (Schuurmans et al., 2005) consists of six 3-item subscales. Items corresponding to the taking initiative (INI; e.g., “How often do you take initiative to get in touch with people who are dear to you?”), investing (INV; e.g., “Do you ensure that you have enough interests on a regular basis [such as a hobby] to keep you active?”), and positive frame of mind (PFM; e.g., “When you have a bad day, how often do you think that things will be better tomorrow?”) subscales were scored on a 6-point Likert scale ranging from never (1) to very often (6). Another 6-point scale, with 1=none, 2=one, 3=two, 4=three or four, 5=five or six, and 6=more than six, was used to score the items corresponding to variety subscale (VAR; e.g., “How many hobbies or activities do you have on a regular basis?”). Items corresponding to the multifunctionality subscale (MUL; e.g., “The activities I enjoy, I do together with others.”) were scored on a 5-point Likert scale ranging from strongly disagree (1) to strongly agree (5). Finally, items corresponding to the self-efficacy subscale (SEF; e.g., “Are you able to have friendly contacts with others?”) were scored on a 5-point Likert scale ranging from I am certain that I cannot (0) to I am completely certain that I can (5). Scores were calculated by recoding the scores to 0–5 or 0–4 (for the 6 and 5-point scales, respectively) and multiplying the items with six options by 4 and the items with five options by 5. After that, subscale scores were determined by taking the average of all items corresponding to each scale and multiplying that score by 5. Hence, subscale scores range from 0 to 100, with higher scores reflecting higher SMA in that dimension. SMAS total scores were calculated by taking the average of all mean subscale scores. Here, higher scores indicated higher overall SMA.

#### Physical Activity

While there are plenty of validated self-report questionnaires on physical activity, most of them are detailed and take a long time to complete. Since we aimed to minimize the burden of participants, we only used a limited number of questions to determine a general measure of physical activity. These were (partially) inspired by the Physical Activity Questionnaire used in the Longitudinal Aging Study Amsterdam (LASA Team, 2020). First, participants were asked to report whether they were sitting in a wheelchair or not. Subsequently, they had to indicate how many hours/min they have been doing light to moderate physical exertion (e.g., walking, cycling, and light household chores) in the past week. Similarly, they were asked to indicate how many hours/min they have been doing vigorous physical exertion (e.g., causing rapid breathing or shortness of breath; making you sweat, such as an intense bike ride, gymnastics/fitness, heavy household chores, etc.) in the past week. An intensity-weighted total physical activity score was subsequently calculated by taking into account the different intensities, using Metabolic Equivalent of Task (MET) scores (with one MET unit reflecting 1kcal per kg body weight per hour). In principle, each activity can be linked to a specific MET score, where energy consumption of light to moderate physical activities ranges from 1.6 to 5.9 METs, and energy consumption of vigorous physical activity is 6.0 MET or more. Here, we used an average MET score of 3.75 for mild to moderate and six for vigorous activities and multiplied those scores by the durations of the two intensity categories. Finally, these scores were summed and corrected (multiplied by) for sitting in a wheelchair or not, with no=1, normal wheelchair=0.8, mechanic wheelchair=0.5, and referred to as PHY.

#### Stringency Index

A measure of strictness of “lockdown style” policies that restricted people’s behavior in the Netherlands at the time of participation was added to control for possible effects of the COVID-19 pandemic on QoL and the included resilience factors. The Stringency Index (SI) of the Oxford COVID-19: Government Response Tracker (OxCGRT)^1^ is a composite measure based on one health system policies indicator (i.e., presence of public info campaigns) and eight containment and closure policies indicators (i.e., closings of schools and universities, closings of workplaces, cancellations of public events, limits on private gatherings, closing of public transport, order to confine to the home, restrictions on internal movement between cities/regions, restrictions on international travel). If participation was divided over multiple days, the average SI of those days was used.

### Data Analysis

#### Overview

##### Main Analyses

Several networks were estimated to explore the interplay between resilience factors and (facets of) QoL. Each network comprised the graphical representation of the relationships (i.e., edges) between the included variables (i.e., nodes). The first set of analyses included the following variables: overall QoL, BC, PAS, SMA, PHY, and Stringency Index (SI). To identify which resilience factors had the strongest contribution on overall QoL reports and could potential improve QoL when being manipulated, both a Gaussian graphical model (GGM) and directed relative importance network were constructed (six nodes). While the former provided us with information on the unique relationships between the nodes, the latter particularly enabled us to explore the potential causal relationships among the individual nodes. The edges in the GGM are undirected and specify the strength of association (positive or negative) between two nodes while taking into account all other nodes (i.e., partial correlations). In the relative importance network, edges are directed and represent the unique contribution of a given node – the regressor – to the prediction of another node – the outcome. The second set of analyses was conducted to establish how the resilience factors interact with the different facets of QoL, and how each factor may promote a specific set of facets. To this end, QoL was split into SAB, AUT, PFF, SOP, INT, and DAD, and similar networks were estimated (11 nodes).

##### Exploratory Analyses

A more exploratory set of analyses was performed by including all the individual facets of the SMAS (i.e., INT, INV, SEF, VAR, MUL, and PFM), in addition to all the other nodes included in the previous networks (15 nodes). Again, we constructed a GGM and directed relative importance network and identified the interplay among those factors.

#### Statistical Analysis

##### GGM Estimation

The *ggmModSelect* algorithm, as included in the qgraph and bootnet R-packages (Epskamp et al., 2012, 2018a), was used to estimate unregularized GGMs. This algorithm searches for an optimal unregularized GGM, by minimizing the (extended) Bayesian information criterion (EBIC; Foygel and Drton, 2010; Isvoranu and Epskamp, 2021). This resulted in non-biased parameters, with the edges representing *partial correlation coefficients*. In our networks, EBIC hypertuning parameter (*γ*) was set to 0, indicating BIC model selection.

Interactions between factors were visualized and interpreted using qgraph (Epskamp et al., 2012), where nodes with stronger associations were positioned at the center of the network and nodes with weaker connections were placed in the periphery, according to the Fruchterman and Reingold (1991) algorithm. The thickness and saturation of the edges signify the magnitude of the partial correlation coefficients, where green and red edges represent positive and negative correlations, respectively. The stability of the edge weights was assessed by using nonparametric bootstrapping (i.e., resampling data with replacement, 1,000 samples; Efron, 1979; Bollen and Stine, 1992; Hastie et al., 2015) and constructing quantile intervals around the edge weights. The narrower the quantile intervals are, the more stable the edge-weight estimates. Second, a *bootstrapped difference test* was performed to identify whether edge weights significantly differed from each other. This was done by taking the difference between bootstrapped values of the edge weight and constructing a quantile interval around each difference score. Edges were deemed significantly different (*p*<0.05) if zero was not in the quantile interval.

##### Relative Importance Network Estimation

To explore potential causal relationships among QoL (facets) and resilience factors, the structure of each GGM was also imposed on a relative importance network estimation. Using the *lgm* metric as provided by the *relaimpo* R software package (Grömping, 2006) and *qgraph* (Epskamp et al., 2012), we constructed several *relative importance networks* (Robinaugh et al., 2014; McNally et al., 2015; Heeren and McNally, 2016; Hoorelbeke et al., 2016; Bernstein et al., 2017). Relative importance was quantified as the proportional contribution in predictability (i.e., amount of explained variance, *R*^2^) attributable to each predictor, ranging from 0 to 1. The resulting networks consisted of two opposing directed edges between each pair of nodes, with the directionality of these edges representing the directionality of the predictions. Through this, we could distinguish the *outstrength* (i.e., the extent to which a certain node predicts the variance of the connected nodes) and *instrength* (i.e., the extent to which variance of a node is predicted by other connected nodes) of each node. We calculated the total out- and instrength for each node (i.e., sum, considering all relationships) and additionally only the direct relationships of the resilience factors with the QoL nodes(s) were considered. This enabled us to better explore the predictive strength (i.e., relative importance as predictor) of each node in influencing the network, as well as how easily a node might be changed by manipulating the nodes directly related to that node.

The stability of the *directed* edges of the relative importance network was assessed by using nonparametric bootstrapping, with quantile intervals being estimated for each directed edge. Subsequently, a quantile interval was estimated around the summed out- and instrength differences of interest (see above). A *bootstrapped difference test* was performed to identify significant differences (*p*<0.05) between the two directed edges of each node-pair, as well as the summed out- and instrength differences of interest per node.

Finally, we established the stability of the directed edges under case-dropping subset bootstrapping. This allowed us to quantify stability in terms of a correlation stability coefficient (CS), the maximum proportion of cases that can be dropped to retain with 95% certainty a correlation of at least 0.7 with the original out and instrength estimations (Cohen, 1977). The higher the CS, the more stable the corresponding strength value.

## Results

### Descriptive Statistics

Visual inspection revealed normal distributions for QoL, BC, PAS, and SMA, but a considerably skewed distribution of the PHY data. Hence, this variable was subjected to the robust *median absolute deviation* outlier detection mechanism (Leys et al., 2013), with a threshold of 2.5, leaving 1,392 participants (520 males, 871 females, one other) that were included in the analyses. Participants had a mean age of 68.7years old (SD: 6.92, range 55–93), and only two of them used a mechanical wheelchair. Participants reported a considerably high educational attainment, with the majority (86%, *N*=1,196) having completed a high education level (Verhage 6 or 7; Verhage, 1964).

Descriptive statistics of the QoL and the resilience factors of interest are reported in Table 1. Graphical results of the (main) stability analyses of the individual (directed) edges can be found in the supplemental results (Supplementary Figures 1–10). In general, the edge weights exhibited relatively small quantile intervals, but some of the absolute edge weights did not differ significantly from one another, indicating that the relative order and contribution (in case of outstrength vs. instrength comparisons) of some edges should be interpreted with caution. The CSs of both the in-and outstrength value were 0.75 for all estimated networks.

**Table 1:** Descriptive statistics of the QoL (facets) and the resilience factors for all participants.

Construct (abbreviation; possible range)	M (SD)	Observed range
Quality of life (QoL; 24–100)	94.6 (9.52)	51–120
Sensory abilities (SAB; 4–20)	17.6 (2.61)	6–20
Autonomy (AUT; 4–20)	15.9 (2.00)	7–20
Past, present, and future activities (PPF; 4–20)	15.9 (2.07)	6–20
Social participation (SOP; 4–20)	15.7 (2.61)	5–20
Death and dying (DAD; 4–20)	14.7 (2.89)	4–20
Intimacy (INT; 4–20)	14.9 (2.92)	4–20
Behavioral coping (BC; 8–32)	21.5 (3.71)	9–32
Positive appraisal style (PAS)^a^	0.03 (0.60)	−1.78–1.72
Self-management ability (SMA; 0–100)	69.2 (11.6)	28.9–98.9
Taking initiative (INI; 0–100)	69.7 (16.7)	20–100
Investment behavior (INV; 0–100)	77.0 (15.6)	20–100
Self-efficacy (SEF; 0–100)	58.6 (16.14)	6.67–100
Variety (VAR; 0–100)	60.8 (16.5)	6.67–100
Multifunctionality (MUL; 0–100)	83.2 (14.9)	25–100
Self-efficacy (SEF; 0–100)	83.2 (14.9)	25–100
Positive frame of mind (PFM; 0–100)	66.0 (17.3)	0–100
Physical activity (PHY)^b^	3,641.6 (2,109.6)	0–9,180
Stringency Index (SI; 0–100)	65.9 (8.4)	56.48–82.41

a *z*-score.

b Composite score: physical activity duration weighted by MET.

### Main Analyses

#### Which Resilience Factor Has the Strongest Contribution to Overall QoL?

**Figure 1:**
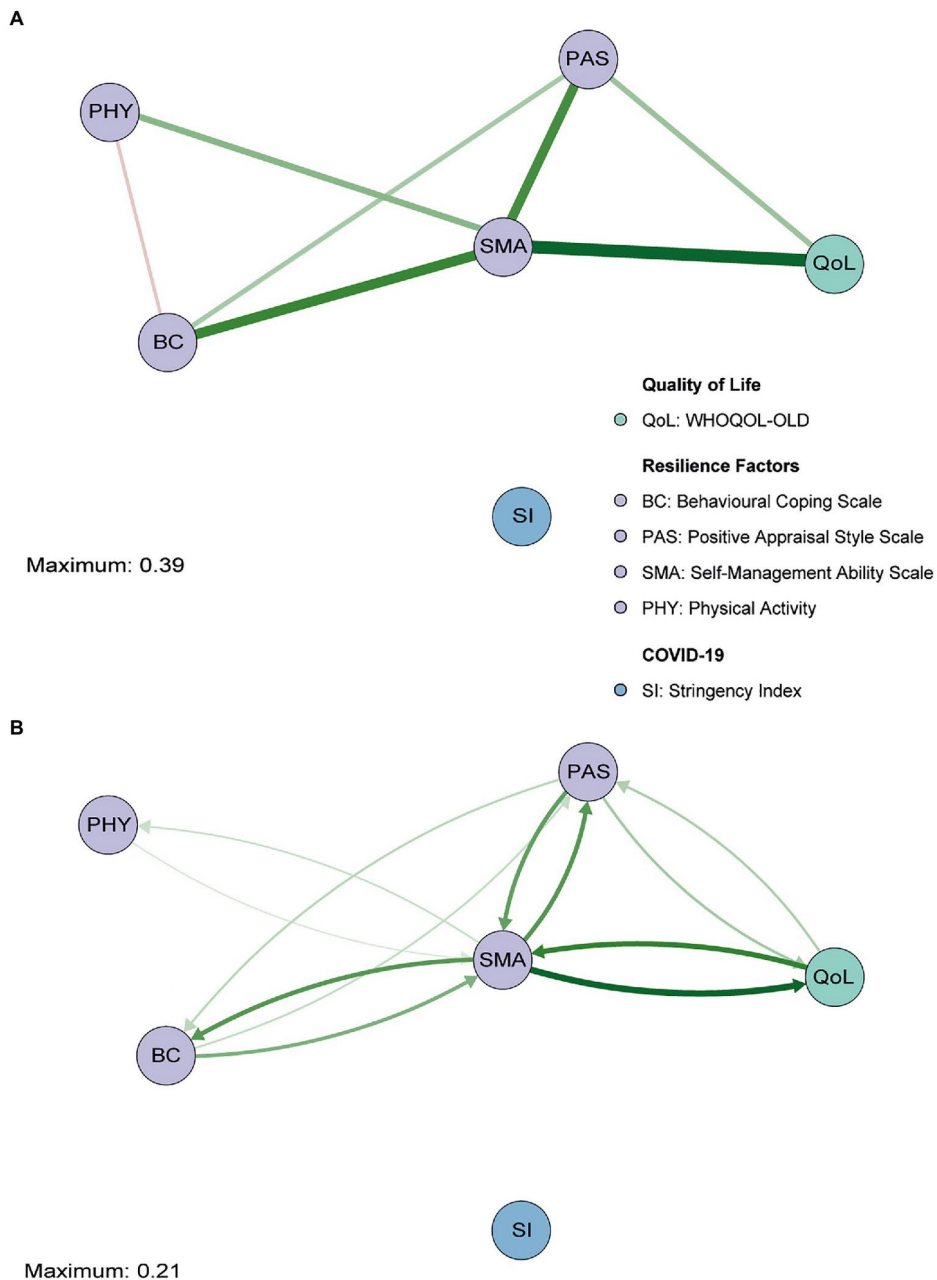
Gaussian graphical model (GGM; **A**) and directed relative importance network **(B)** of overall quality of life (QoL; green), the resilience factors (purple), and the stringency index (blue). The maximum value represents the highest edge weight included in the network.

The first GGM highlighted that all nodes were directly or indirectly connected to each other, except for SI (Figure 1A). This indicates that the Stringency Index did not influence QoL, nor any other factors. QoL was directly connected to PAS and SMA, with the latter being positioned at the center of the network and showing the strongest relationship with QoL (*r*=0.39 vs. *r*=0.15 of PAS, *p*<0.05; Supplementary Figures 1, 2). Moreover, the relationships of both BC (*r*=0.31) and PAS (*r*=0.29) with SMA, as well as their own unique association (*r*=0.14), were fairly strong. The partial correlation between SMA and PHY (*r*=0.18) also indicated an indirect relationship between QoL and PHY. Finally, contrary to our expectations, BC was negatively related to PHY (*r*=−0.09), although relatively weakly (all *p*’s<0.05) with the edge set to zero relatively often (35%) compared with all other edges (<1%) with bootstrapping (Supplementary Figures 1, 2).

**Figure 2:**
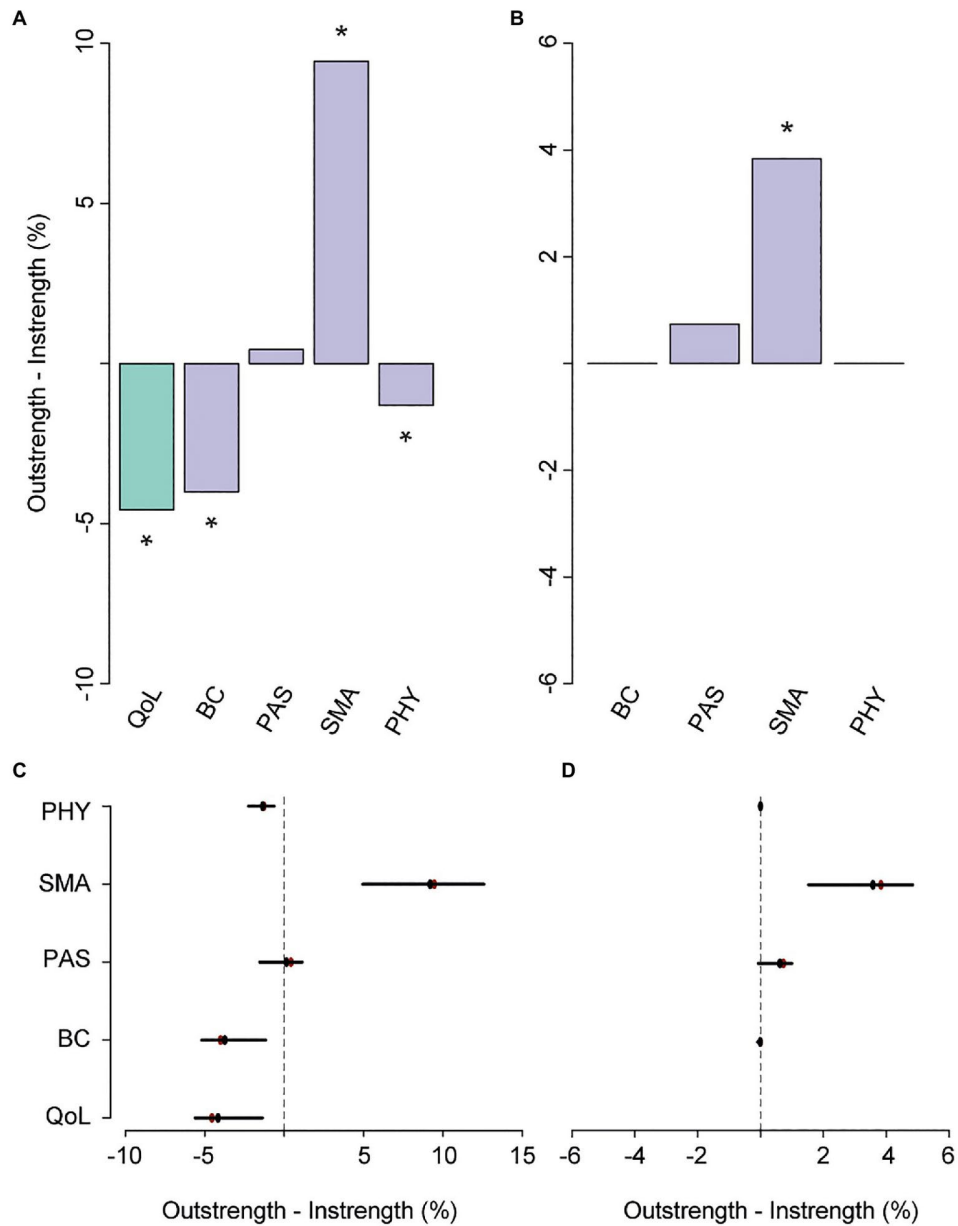
The difference between overall outstrength and instrength of the nodes in the primary network **(A)** and the difference in outstrength and instrength of the relationships between the resilience factors and QoL only **(B)**. Colors of the bar charts correspond to the nodes in the network in Figure 1. In plots (**C**,**D**), the bootstrapped mean is depicted in black and the sample mean in red. ^*^p<0.05; nodes with quantile intervals containing zero are deemed to have an insignificant instrength and outstrength difference.

The *relative importance network* revealed that SMA and PAS together accounted for 27.8% of the variance in QoL (instrength), while QoL only explained 23.3% (outstrength) of the variance of these resilience factors (Figure 1B; see Figures 2A,C for difference plots; *p*<0.05). SMA appeared to be the main hub in the network, exerting a large influence on all the other nodes (52.4%). The total outstrength value was even significantly larger than the total instrength value (42.9%, *p*<0.05; Figures 2A,C; see Supplementary Figures 3, 4 for individual edges). PAS had the second largest outstrength value (25.2%), but this was relatively similar to its instrength value (24.7%, *p*>0.05). These results remained present after excluding the outstrength of SMA and PAS on the other resilience factors (Figures 2B,D). Finally, we observed a larger instrength than outstrength value of both BC and PHY (Figures 2A,C), suggesting that SMA (and PAS, in case of BC) exerted a stronger influence on those factors than vice versa (both *p*’s<0.05; and see Supplementary Figures 3, 4 for individual edges).

Hence, these networks suggest that manipulating SMA or PAS could result in improved QoL reports. Since SMA had the highest relative importance as a predictor of QoL (compared with PAS, *p*<0.05, Supplementary Figure 4), which was significantly higher than the corresponding instrength value, this resilience factor seemed to be most opportune to direct interventions or individual efforts on when trying to improve overall levels of QoL.

#### How Are the Resilience Factors Related to Different Facets of QoL in Older Adults?

**Figure 3:**
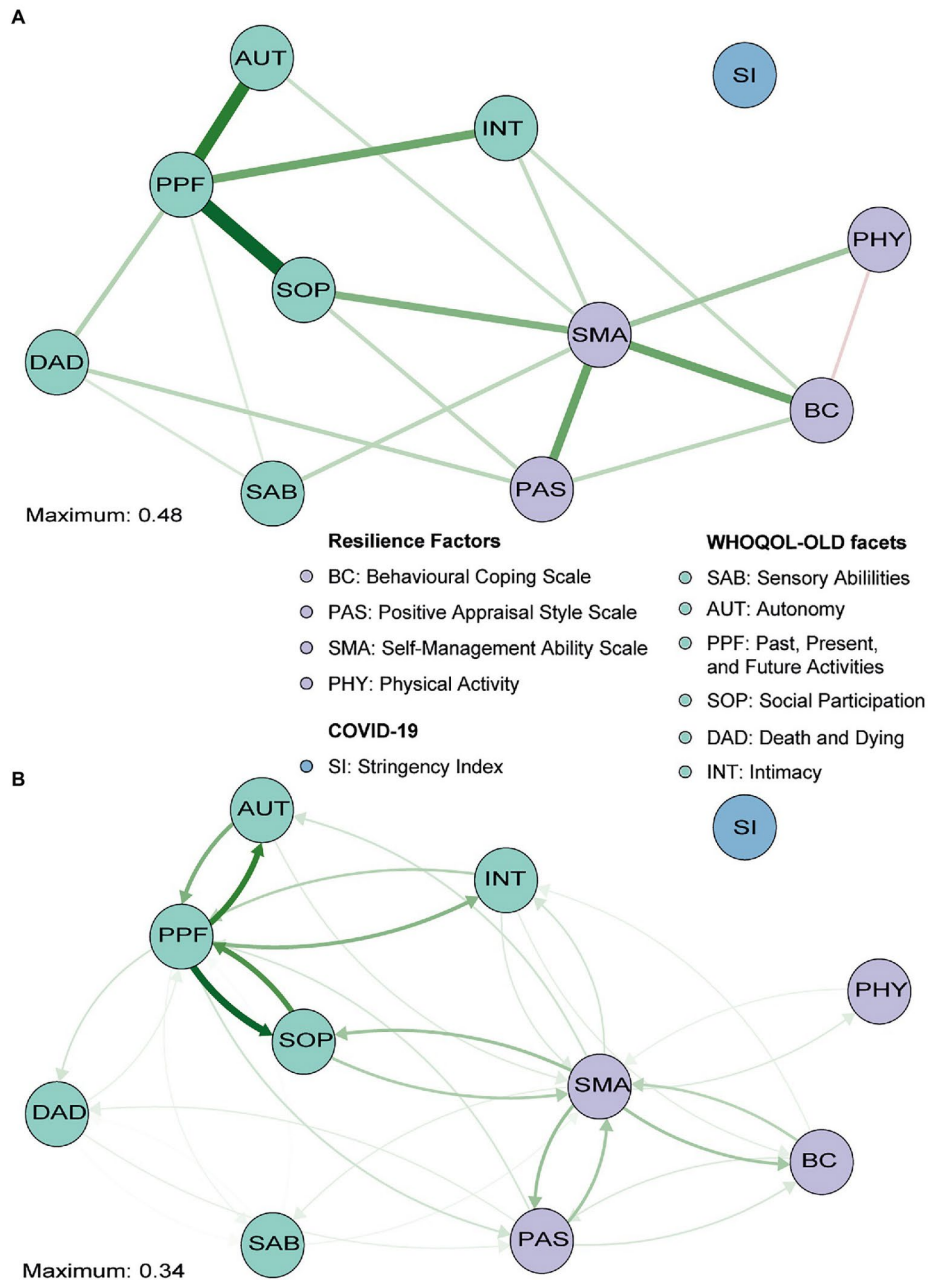
Gaussian graphical model (GGM; **A**) and directed relative importance network **(B)** of individual facets of QoL (green), the resilience factors (purple), and the stringency index (blue). The maximum value represents the highest edge weights included in the network. Note that the direct relationship from positive appraisal (PAS) to past, present and future activities (PPF) is covered by the social participation (SOP) node and that PAS is thus not directly related to SOP (only indirectly *via* PPF).

The GGM with the individual facets of QoL being considered separately also showed that only the SI was not (in)directly connected to the other nodes (Figure 3A). As expected, we found similar relationships between PAS, SMA, BC, and PHY compared with the overall QoL network (Supplementary Table 1). Edge weights of the relationships among all facets of QoL are shown in Supplementary Table 2. In addition to those clustered connections, some interesting relationships between the resilience factors and the individual facets of QoL were found (see Supplementary Table 3 for edge weights). SMA appeared to be especially strongly connected to the SOP facet of QoL and to a lesser extent to SAB, INT, and AUT (all lower than SOP: *p*’s<0.05; see Supplementary Figures 5, 6). A direct relationship between SMA and both DAD and PPF was not observed, but PAS appeared to be related to both facets (with similar strengths, *p*>0.05). Finally, while BC was not directly related to overall QoL in the primary network, a relationship with the INT facet specifically was observed in this network (similar to SMA – INT, *p*>0.05, but more stable: 7% vs. 17% of bootstraps set to zero; Supplementary Figures 5, 6). This reveals that the QoL facets are directly related to different resilience factors.

**Figure 4:**
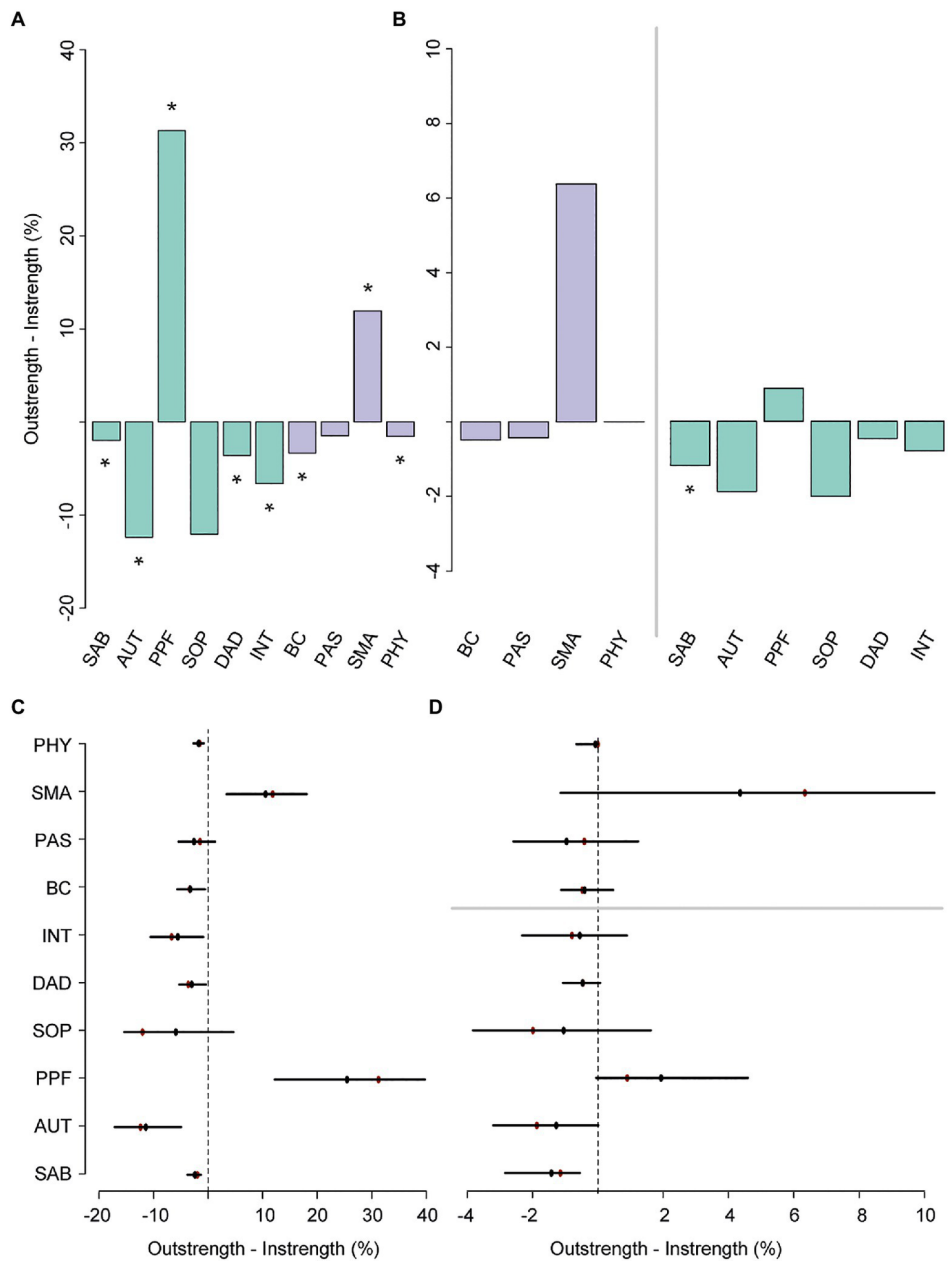
The difference, including bootstrapped quantile intervals, between total outstrength and instrength of all the nodes in the secondary network **(A,C)**, and the difference in total outstrength and instrength of the relationships between the resilience factors and QoL facets only (**B**, left; **D**, top), and the relationships between the QoL facets and the resilience factors (**B**, right; **D**, bottom). Colors of the bar charts correspond to the nodes in the network in Figure 3. In plots (**C**,**D**), the bootstrapped mean is depicted in black and the sample mean in red. ^*^p<0.05, nodes with quantile intervals containing zero are deemed to have an insignificant instrength and outstrength difference.

In line with earlier findings, the directed relative importance network (Figure 3B) revealed that SMA had a relatively high total outstrength value (57.1%) compared with its instrength value (45.2%, *p*<0.05; Figures 4A,C; see Supplementary Figures 7, 8 for individual edges). PAS seemed to have a relatively larger instrength (27.0%) than outstrength value (25.5%), although this was not significant (*p*>0.05). Subsequent calculations that only included the relationships with the QoL facets, and not with other resilience factors, revealed that SMA again exerted a larger total influence on the facets of QoL (27.5%) than vice versa (21.1%), although not significantly so, due to a relatively unstable estimate of the difference (large quantile interval; Figures 4B,D). For BC, the total outstrength value was lower than the instrength value (2.9% vs. 3.4%, *p*<0.05), whereas for PAS no difference was observed (8.6% vs. 9.0%, *p*>0.05). Indeed, both the total instrength -and outstrength value of PFF were considerably high, with the latter in particular (59.7 vs. 91.0%, *p*<0.05). However, when excluding the relationships of PPF with the other QoL facets, the outstrength value (6.0%) was relatively similar to the instrength value (5.1%; *p*>0.05; Figures 4B,D). This suggest that PPF is an important facet of QoL, as it impacted many other QoL facets, but that PPF and PAS have similar relative importance as a predictor.

Thus, while overall QoL reports (first set of analyses) can possibly be enhanced by directing interventions or individual efforts on SMA, and PAS to a lesser extent, the second set of analyses further revealed that the selection of the most appropriate resilience factor (partially) depends on the goal. That is, boosting SMA could potentially result in improved reports on the SAB, SOP, INT, and AUT subscales. However, intervening on PAS may be particularly relevant when aiming to decrease worries about DAD or improve satisfaction about achievements in life and at things to look forward to (PPF).

### Exploratory Analyses

#### How Do Different SMAs Relate to the QoL Facets and Other Resilience Factors?

**Figure 5:**
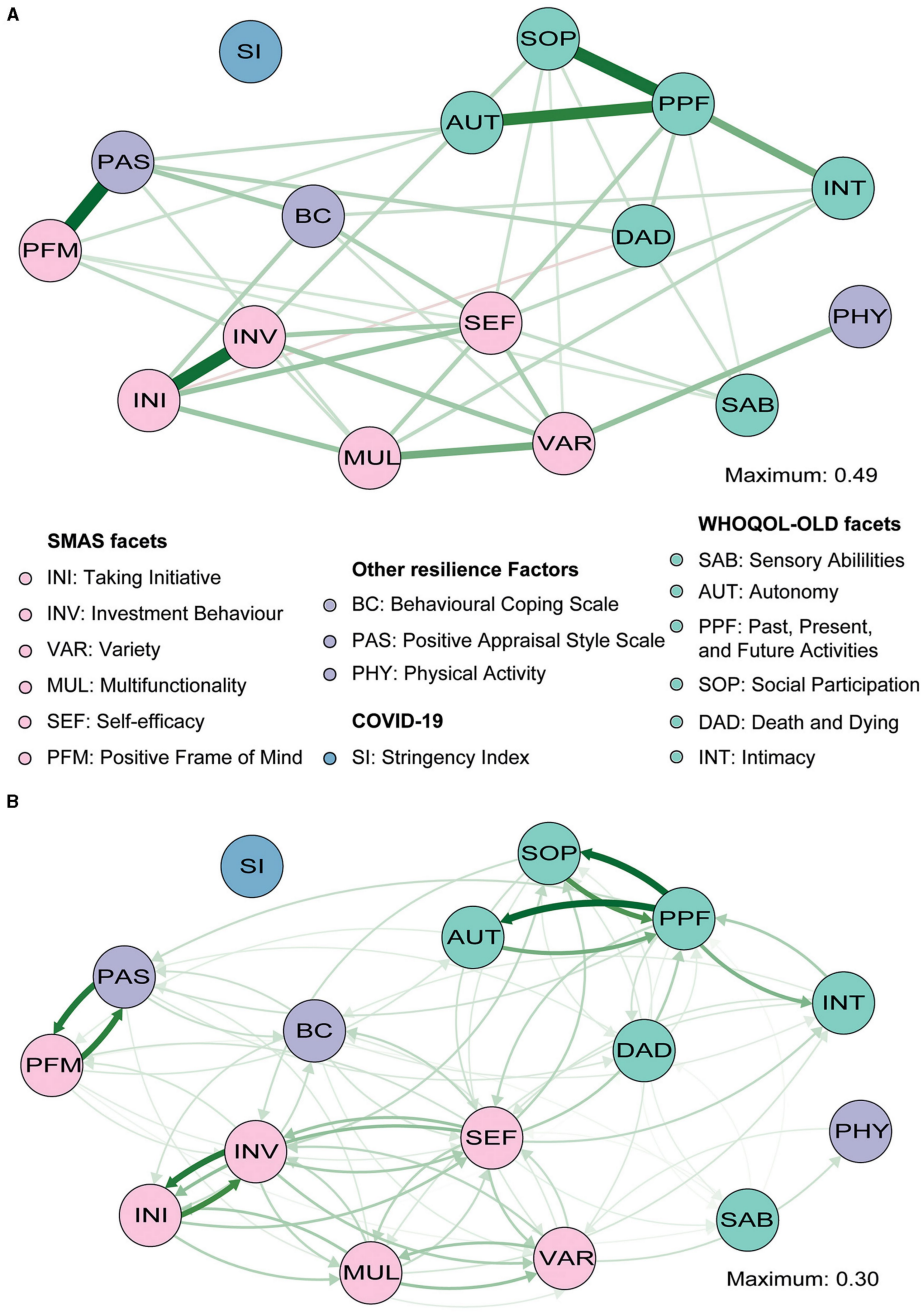
Gaussian graphical model (GGM; **A**) and directed relative importance network **(B)** of individual facets of QoL (green), the facets of the SMAS (pink) and other resilience factors (purple), and the stringency index (blue). The maximum value represents the highest edge weights included in the network.

**Figure 6:**
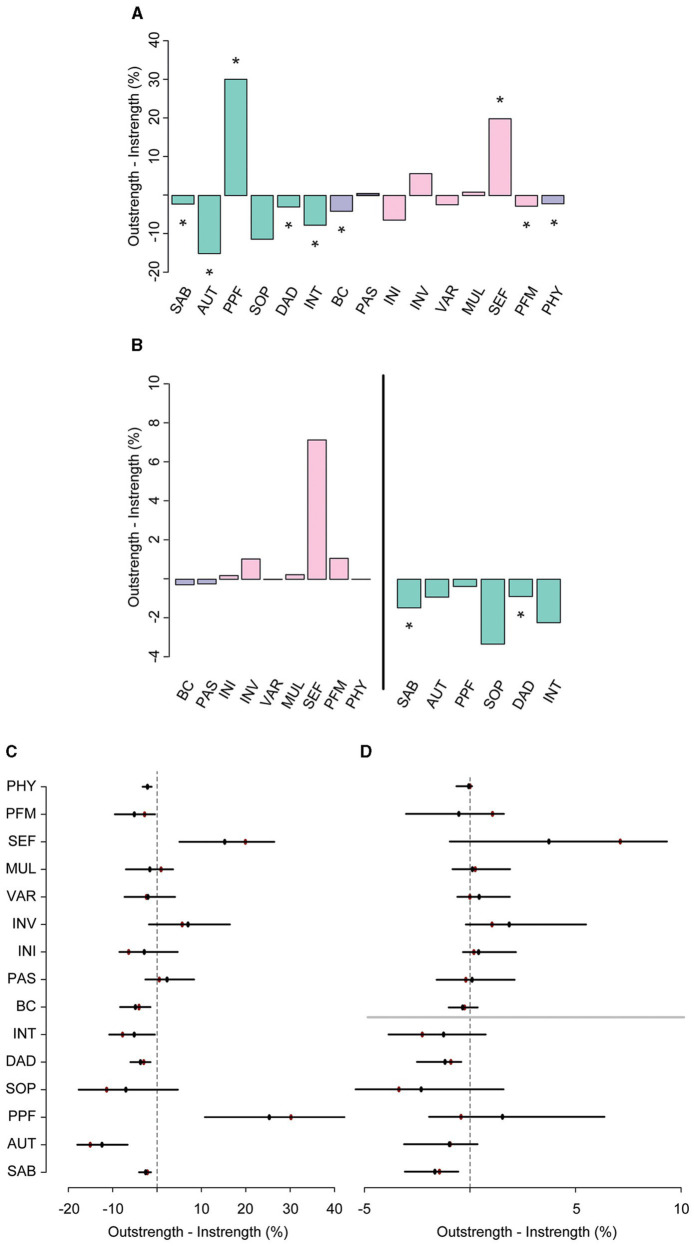
The difference between overall outstrength and instrength of the nodes in the third network **(A)**, and the difference in outstrength and instrength of the relationships between the resilience factors and QoL facets only (**B**, left), and the relationships between the QoL facets and the resilience factors (**B**, right). Colors correspond to the nodes in the network in Figure 5. In plots (**C**,**D**), the bootstrapped mean is depicted in black and the sample mean in red. ^*^p<0.05, nodes with quantile intervals containing zero are deemed to have an insignificant instrength and outstrength difference.

While earlier analyses suggest that especially SMA is an important factor, it remains unclear what specific self-management abilities are crucially involved. Through exploratory analyses, we aimed to establish whether there are substantial differences in the importance of the six SMA facets included in the SMAS. A third GGM again highlighted that almost all nodes were (in)directly connected to each other and revealed similar associations between the QoL facets (Figure 5A; Supplementary Table 4). Edge weights of connections among nodes of the individual facets of the SMAS, as well as the other resilience factors, are reported in Supplementary Table 5. Not surprisingly, we observed a particularly strong connection between positive appraisal style (PAS) and the PFM facet of the SMAS, and a relatively weak (and less stable) connection between PAS and MUL (*p*<0.05, Figure 5A; Supplementary Figures 9, 10). Both relationships had similar instrength and outstrength values (*p*’s>0.05; Figure 5B) and influenced each other equally. VAR was directly related to physical activity (PHY), with VAR exerting a larger influence on PHY than vice versa (4.7% vs. 2.6%; *p*<0.05; Figures 6A,C). This builds on the relationship between SMA and PHY observed in the previous networks and suggests that engaging in physical activity may indirectly enhance QoL, as it helps one to ensure a variety of external resources (e.g., maintaining several friendships) to achieve certain goals in life, but that ensuring such a variety of resources may also strongly (and even to a larger extent than vice versa) promote physical activity. In addition to the previously established connection with PAS, behavioral coping (BC) was also directly related to the VAR (not consistently, 30.6% of the bootstraps set to zero), INI, and SEF facets of the SMAS in this network. Taking all these edges together, the total instrength value of BC was higher compared with the outstrength value (19.0 vs. 15.2%, *p* > 0.05). This is in line with earlier suggestions that boosting (specific) SMAs may not only improve QoL, but also other resilience factors (e.g., BC or PHY) that can indirectly further enhance QoL.

Of all the SMAS facets, SEF had the most direct connections with facets of QoL (i.e., SAB, SOP, INT, PPF; Figure 5A and Supplementary Table 6) and the largest total outstrength-instrength difference (69.7%−49.8%; *p*<0.05; Figures 5B, 6A,C), even when excluding the relationships with other resilience factors (22.8%−15.7%). However, in the latter situation, the estimation of the difference was relatively unstable, resulting in a large quantile interval that contained zero (*p*>0.05; Figures 6B,D). The connection of SEF with the PPF facet (7.7 vs. 6.4%, *p*>0.05) was of particular interest, since PPF was not directly related to overall SMA in the second GGM. Moreover, this connection appeared to be stronger than the edge between PPF and PAS, although not significantly (*p*>0.05; Supplementary Figures 9, 10). These exploratory findings suggest that, potentially, when aiming to improve the PPF facet of QoL, one should focus on enhancing one’s belief in the competence to achieve certain goals in life, rather than PAS. Due to the considerably high total outstrength value of PPF on other facets of QoL (79.0 vs. 48.4% instrength, *p*<0.05), this may also be an excellent strategy to indirectly enhance AUT (29.7%), SOP (29.3%), INT (14%), DAD (4.3%), and SAB (1.7%), and thereby QoL as a whole. Several other positive (and some negative) relationships between the individual SMAs and QoL facets were observed as well, although most of them were considerably unstable (see Figures 5, 6 and Supplementary Figures 9, 10).

In sum, these exploratory analyses again highlight that the selection of the most appropriate resilience factor to manipulate depends on the quality of life (QoL) facet one aims to promote. Most interestingly, it seems that targeting the self-efficacy facet of the SMAS can potentially have the strongest effect on overall QoL. This facet was strongly related to the past, present, and future (PFF) activities facet of QoL, which in turn exerts a large influence on multiple other QoL facets.

## Discussion

The aging process is accompanied by challenges and transitions that can lead to significant changes in QoL if these are not adequately managed and regulated. The present study adopted a network approach to investigate the interplay between key resilience factors and QoL (as an outcome measure of resilience) in later life. Our expectation to find positive relationships between (facets of) QoL and our resilience factors of interest, either directly or indirectly, received strong support. We first present a brief summary of the findings, followed by more in-depth discussion of the observed relationships.

Most fundamentally, we provide evidence to suggest that individual differences in SMA and (to a slightly lesser extent) PAS play a crucial role in predicting QoL. The first set of analyses revealed that SMA was the most influential resilience factor, not only having direct relationships with QoL, but also serving as an important connecting link between QoL and other positive resources (e.g., behavioral coping, physical activity). Moreover, both SMA and PAS exhibited a high relative importance as predictor of QoL (i.e., outstrength on QoL), albeit that only for SMA this was higher than its instrength.

The relationship of SMA with QoL was driven by various underlying associations with multiple facets of QoL, includingsensory abilities (SAB), social participation (SOP), autonomy (AUT) and intimacy (INT). Moreover, while PAS appeared to influence overall QoL to a lesser extent than SMA, it was strongly connected to specific facets, namely DAD and PPF. Therefore, manipulation of PAS may be an effective pathway for decreasing worries about death and dying and improving satisfaction about achievements in life and at things to look forward to. However, exploratory analyses revealed that specifically targeting the self-efficacy (SEF) facet of the SMAS could also be a promising strategy to improve reports on PPF. Considering the large influence that PPF exerts on other QoL facets, SEF may be an excellent target for interventions or individuals’ own efforts to promote overall QoL.

### Main (and Exploratory) Findings

Our main finding that SMA is strongly associated with (multiple facets of) QoL (especially SOP) extends earlier demonstrations of positive associations between SMA and well-being and other health indicators (e.g., frailty, loneliness) at old age (Schuurmans et al., 2005; Cramm et al., 2012a,b, 2014; Nieboer et al., 2020). Moreover, our finding is in line with Cramm and Nieboer (2017) that successfully increased QoL by boosting SMA. The connections of SMA with other resilience factors, with SMA having a high relative importance as predictor of these factors, also suggest that a strong capacity to take care of one’s life and external resources indirectly improves QoL by promoting other factors that can enhance QoL. Individuals with a strong SMA may have a richer repertoire of cognitive or behavioral coping abilities, but the effective implementation of SMAs may also provide opportunities or optimal circumstances to effectively employ specific coping strategies (e.g., many BC strategies require involvement of others; positively appraising a situation may be easier when one generally feels in control of their own life). As such, SMA may play an important role in the reinforcing spirals (or feedback loops) among the resilience factors. This is in line with the idea that resilience should be represented as a combination of protective factors that do not function in isolation, but are interconnected and (potentially) strengthen one another (e.g., Fritz et al., 2018, 2019; Scheffer et al., 2018; Brinkhof et al., 2021).

In addition to this main finding, the exploratory analyses on the individual SMAs revealed that SEF was relatively strongly connected to the PPF facet of QoL. This implies that the ability to gain and maintain a belief in one’s personal competence, control and self-efficacy in achieving certain goals in life at old age contributes to the extent to which one is satisfied with past, present and future activities. Potentially, individuals with strong self-efficacy beliefs are more likely to undertake the activities and efforts needed to achieve their goals (Steverink et al., 2005), and as a consequence they are more satisfied with achievements in life and things to look forward to. Since PFF has a large outstrength on other facets of QoL, SEF may be a critical target for interventions aiming to improve overall QoL. Indeed, aging involves transitions and changes that introduce new challenges and uncertainties, which can undermine individuals’ self-efficacy beliefs (Steverink et al., 2005; Nieboer et al., 2020). This may be due to a sudden physical limitation, or fewer opportunities for social contacts (e.g., interaction with colleagues, physical exercise within a group) and skill development (e.g., learning new things in a working environment), as well as increasing experiences of loss and failure. Hence, building interventions that help individuals to promote SMA, and self-efficacy in particular, thereby reducing potential declines in QoL (and wellbeing), is highly important.

One of the few existing interventions that has been developed for this purpose is the self-management of wellbeing (SMW) intervention, tested in different formats (individual, group and self-help; Schuurmans, 2004; Frieswijk et al., 2006; Kremers et al., 2006; see Goedendorp and Steverink, 2017 for comparison). However, this intervention is high intensive, involving multiple (5–6) session (of 1–2.5 h). To improve the accessibility for older adults, it may be useful to explore possibilities for low intensity interventions that focus on teaching individuals how to successfully adjust their behavior in accordance with internal or external demands and challenges, thereby fostering their belief in the personal competence to achieve life goals in general. Promoting the use of the strategic planning technique of implementation intentions could be useful for this purpose (Gollwitzer, 1999).

Although to a lesser extent than SMA, PAS was also strongly and directly associated with QoL. This is in line with the notion that PAS is protective for mental health (Kalisch et al., 2015) and with earlier research showing that the use of cognitive (emotion regulation) coping can contribute to positive (mental) health outcomes at old age specifically (Garnefski et al., 2002; Kraaij et al., 2002; Garnefski and Kraaij, 2006b; Swift et al., 2008; Nowlan et al., 2015, 2016). Positive reappraisal most frequently emerged as adaptive strategy in these studies. It involves finding a positive meaning within a negative situation and has been shown to be effectively implemented by older adults (Shiota and Levenson, 2009; Hall et al., 2010; Nowlan et al., 2015). Hence, this strategy seems to be a good starting point for interventions intended to improve overall QoL (e.g., mindfulness; Garland et al., 2009), but possibly also to improve satisfaction with achievements in life and things to look forward to (PFF) and to reduce concerns, worries, and fears about DAD, specifically. Indeed, previous studies have found positive associations with overall life satisfaction (Swift et al., 2008; Windsor, 2009), and the few existing reports on coping with death anxiety have claimed that strategies involving positive re-evaluation or re-organization of thinking should be a main component of treatment (Cicirelli, 2003; Purer and Walker, 2008). Since our research points toward a *general* benefit of a PAS, we suggest future studies to examine the intervening potential of this generic tendency as well.

Our results also suggest that PAS contributes more strongly to QoL than BC. BC was also positively related to QoL, but this was mediated by PAS (and SMA), which exhibited a larger relative importance as predictor of BC than vice versa. This is in line with earlier suggestions that a focus on changing one’s emotional response, rather than managing the situation that elicited them, is more adaptive at old age, since many challenges and event in late life are negative and often unalterable (Folkman and Lazarus, 1980; Cheng, 2001; cf. Nowlan et al., 2015’s perspective on positive reappraisal). Moreover, considering the fact that social networks tend to decline with age (Wrzus et al., 2013) and that many items of the BCS reflect socially supported coping (i.e., requiring the presence or active involvement of others; Nahlen Bose et al., 2015; Ingrand et al., 2018), BC may also be less favorable in later life. In line with this, the observed relationship between BC and the INT facet of QoL may be explained by the fact that the perceived level of intimacy is inherently associated with the number of people someone can call on in difficult situations. As such, BC strategies may only be successfully implemented by those already experiencing high levels of INT, rendering BC ineffective as strategy to improve levels of intimacy. Accordingly, it has been suggested that aging is accompanied by a general shift from the use of BC, centered on problems and seeking of assistance, to cognitive- and emotion-focused coping (Folkman et al., 1987; Blanchard-Fields and Irion, 1988; Blanchard-Fields et al., 1995; Watson and Blanchard-Fields, 1998; Garnefski et al., 2001; Chen et al., 2018; Ingrand et al., 2018). Still, the effectiveness of BC may ultimately also differ between aging individuals, with different sizes of social networks one can resort to, which should be addressed in future studies.

Our findings provide relatively strong support for our hypothesis that physical activity has a positive effect on QoL (e.g., Windle et al., 2010), either directly or by promoting other resilience factors (e.g., Ávila et al., 2018). That is, PHY was positively associated and linked to SMA, and therefore indirectly to QoL, but the relative importance as a predictor of SMA was low in comparison to its instrength (from SMA). This implies that SMA mediated the relationship between PHY and QoL, but that the contribution of SMA on PHY was larger. This is in line with the idea that strengthening resilience can improve the adherence to exercise behaviors (Resnick and Inguito, 2011). Interestingly, our exploratory analyses revealed that the relationship between PHY and SMA was driven by the variety subscale specifically. This implies that having a variety of external resources to achieve a certain life goal, such as engaging in multiple hobbies or in versatile volunteer activities, or having a diverse network of friends or engaging in various different group activities, can promote regular participation in physical activities. Indeed, having multiple hobbies inherently increases the likelihood that at least one of those involves physical exercise. In addition, one’s social connections can play a vital role in promoting health-oriented behaviors, and having a wide circle of friends may encourage physical activity by providing support and companionship (Smith et al., 2017; Schlenk et al., 2021). In turn, physical activity, particularly group exercise classes and team sports, is likely to foster additional social interactions and personal growth that may contribute to one’s ability to ensure a variety of external resources. Thus, our results suggest that engaging in physical activities can positively shape areas of an individual’s life beyond the physical health, and that enhancing one’s ability to acquire and maintain a variety of resources may greatly enhance overall QoL, as well as engagement in physical activity specifically. The fact that PHY had no direct associations with QoL remains surprising. A possible explanation may be that the current measure of PHY was too general and did not pick up decisive differences among individuals. This has been shown to be a general pitfall of self-report measures of physical activity (Prince et al., 2008), which emphasizes that cautious interpretation is warranted.

The lack of an effect of the stringency of the COVID-19 policies on QoL may be explained by the fact that our study took place during the second wave of the pandemic. Previous studies that were conducted during the initial phase, or its aftermath (summer 2020), have reported either negative (O’Hara et al., 2020; Lee et al., 2021a; Voss et al., 2021), or positive effects on individuals’ mental health (Kim and Jung, 2021; Lee et al., 2021b; Long et al., 2021). Hence, current findings may indicate that, over time, individuals became more resistant to fluctuations in policies and restrictions, possibly because they had become accustomed to the uncertainty and frequent policy changes. Another factor that could explain the discrepancy between the current and aforementioned reports is that we evaluated fluctuations within one country, while others looked at differences between countries (with more or less stringent measures). Interestingly, one previous finding that is in accordance with current observations is that of Ding et al. (2021), who found that after adjusting for *personal risk factors*, a more stringent governmental policy response was not associated with increased anxiety and depression symptoms during the lockdown. Clearly, taken together with the present findings, this suggests that it is important to evaluate multiple factors at once, yet the impact of the stringency index on (outcome based) resilience warrants further in-depth investigation.

### Limitations and Future Directions

Since our analyses relied on cross-sectional data, we could not confirm causal relationships. Indeed, the networks provided clues as to how the resilience factors influenced one another and (facets of) QoL specifically, but intervention studies or other experimental manipulations are required to determine the true intervening potential of these factors. Therefore, the revealed patterns of associations, and hypotheses derived from these, may help guide future research (Epskamp et al., 2018b).

Moreover, the fact that our research took place during the COVID-19 pandemic may have influenced our findings in different ways. We show that fluctuations in COVID-19 policy strictness were too small to affect our measures, but additional research is needed to establish the full impact of the COVID-19 pandemic itself. That is, since our study only included data that were collected during COVID-19, it remains unclear whether a different pattern of associations would emerge if we would include data from after the brunt of the pandemic. Natural variation among individuals in physical activity may have been reduced, due to the restrictions adopted by the Dutch government that limited the opportunities and possibilities to engage in physical activity. Consequently, the (indirect) associations between physical activity and QoL could been affected. Similarly, the implementation of SMAs that focus on managing one’s external resources in a world that is dominated by social restrictions may have been compromised, possibly resulting in different association patterns or at least a dampening of relationships. For example, there were severe restrictions on gathering which in turn limited opportunities to socialize (play sports, go out for dinner, etc.).

One other important constraint to be aware of is the fact that our sample is represented by a predominantly highly educated, native-born group of older adults. This may impact the generalizability of our results, as both education level and migration history have been shown to affect (outcome based) resilience among older adults in a number of previous studies (Lund et al., 2018; Klokgieters et al., 2020; Szabó et al., 2020). Hence, we encourage researchers to perform similar studies to reveal whether the pattern of associations of minority groups approximates the one observed in the current study. Such attempts may require careful considerations of reducing certain barriers (e.g., online, language) that may hold individuals back from participating, for instance by using translated, paper-and-pencil versions.

Further investigation will also be needed to establish whether age can moderate associations among the resilience factors and QoL (facets). Indeed, with advancing age, individuals face an increasing amount of challenges that have to be regulated and managed. This places a greater demand on their capacity for resilience, potentially putting the oldest individuals at higher risk to reach a certain tipping point beyond which they will experience considerable declines in QoL. On the other hand, research has also shown that old age is associated with more beneficial outcomes in the face of adversity (Wilson et al., 2020; Fuller and Huseth-Zosel, 2021; Lind et al., 2021), in agreement with the notion that the accumulation of life events leads to an enlarged repertoire of knowledge and (coping) skills that can help the oldest individuals to deal with the challenges they are confronted with (Norris and Murrell, 1988; Seery et al., 2010; Crane et al., 2019). While the controversial question on the impact of age was beyond the scope of the current study, this important issue will need to be addressed in future work (within our own lab, for one).

Despite these limitations and constraints, the present study widens our understanding of the role of multiple resilience factors in predicting QoL as an outcome of resilience. Most fundamentally, our patterns of associations emphasize the complex and emergent nature of resilience (Brinkhof et al., 2021), showing that resilience in later life arises through interactions among several components, thereby impacting QoL jointly and possibly in a self-reinforcing way (Fritz et al., 2018, 2019; Scheffer et al., 2018). This highlights the importance of investigating the role of resilience factors simultaneously (in complex systems), rather than individually. Future work in our group will focus on improving our comprehension of these complex interactions by evaluating the role and contribution of a wide-ranging pallet of risk factors, in addition to multiple other resilience factors, as well. Adding those factors might change the structure of the network, resulting in a more thorough picture of the contribution of each node. Current relationships between resilience factors and QoL will likely still be obtained (e.g., PAS-QoL), only the pathways through which each factor influences QoL may be more branched than we can currently identify. Moreover, while the contribution of physical activity on the WHOQOL-OLD reports seems relatively low as compared to the other factors included in the current networks, its effects on more health-related QoL measures (e.g., SF-36) may be much stronger. By including both risk and protective factors, we can improve our understanding of the role of favorable and less favorable characteristics (e.g., low SES or availability of green space) or predispositions (e.g., history of depression, anxiety, and low self-esteem) within the overall network, and may, for instance, reveal interesting differences in association patterns between those that have experienced specific life events (such as the loss of a spouse) and those who are relatively unscathed.

## Concluding Remarks

Summarizing, this study contributes to our understanding of the interplay between factors that underpin resilience in later life. We have provided evidence to suggest that SMA and, to a lesser extent, PAS are most crucially involved in the realization and maintenance of high levels of QoL, and building interventions targeting these factors therefore seems most promising when trying to improve QoL. Teaching older adults how they can successfully adjust their behavior to achieve specific life goals, thereby promoting self-efficacy beliefs, may be an excellent starting point for interventions. However, the appropriate set of resilience factors to manipulate may ultimately depend on the facet of QoL that one intends to improve. These findings can aid future studies in determining specific strategies that can help older adults to gain control of their own lives, enabling them to maintain the functional ability and competence that is vital for wellbeing and QoL at old age.

## Data Availability Statement

The datasets presented in this article are not readily available because the datasets used and/or analyzed for the current study will only be made publicly available after completion of the overarching project, and will until that time only be available from the corresponding author on collaboration basis upon reasonable request. Requests to access the datasets should be directed to LB, l.p.brinkhof@uva.nl.

## Ethics Statement

The studies involving human participants were reviewed and approved by the Local Ethics Committee of the University of Amsterdam (2020-DP-12556) and was conducted in accordance with relevant laws and institutional guidelines. The patients/participants provided their written informed consent to participate in this study.

## Author Contributions

LB, KRR, JM, SW, and HK conceived the current study. LB, KRR, and JM conceived the corresponding network analytical framework. LB implemented the study. LB and KH performed the statistical analysis. All authors contributed to the article and approved the submitted version.

## Funding

This research is part of the “Active and Healthy Aging” project, which was funded by the Centre for Urban Mental Health, a Research Priority Area at the University of Amsterdam.

## Preprint

The preprint of this manuscript can be found at https://psyarxiv.com/6vmz9/.

## Conflict of Interest

The authors declare that the research was conducted in the absence of any commercial or financial relationships that could be construed as a potential conflict of interest.

## Publisher’s Note

All claims expressed in this article are solely those of the authors and do not necessarily represent those of their affiliated organizations, or those of the publisher, the editors and the reviewers. Any product that may be evaluated in this article, or claim that may be made by its manufacturer, is not guaranteed or endorsed by the publisher.

## Publisher’s Note

All claims expressed in this article are solely those of the authors and do not necessarily represent those of their affiliated organizations, or those of the publisher, the editors and the reviewers. Any product that may be evaluated in this article, or claim that may be made by its manufacturer, is not guaranteed or endorsed by the publisher.

## Footnotes

^1^https://www.bsg.ox.ac.uk/research/research-projects/coronavirus-government-response-tracker
