# Corrigendum: The interplay between quality of life and resilience factors in later life: a network analysis

**DOI:** 10.3389/fpsyg.2023.1264753

**Published:** 2023-08-08

**Authors:** Lotte P. Brinkhof, Karoline B. S. Huth, Jaap M. J. Murre, Sanne de Wit, Harm J. Krugers, K. Richard Ridderinkhof

**Affiliations:** ^1^Department of Psychology, Faculty of Behavioural and Social Sciences, University of Amsterdam, Amsterdam, Netherlands; ^2^Centre for Urban Mental Health, University of Amsterdam, Amsterdam, Netherlands; ^3^Amsterdam Brain & Cognition (ABC), University of Amsterdam, Amsterdam, Netherlands; ^4^Department of Psychiatry, Amsterdam University Medical Centre, Amsterdam, Netherlands; ^5^Faculty of Science, Swammerdam Institute for Life Sciences, University of Amsterdam, Amsterdam, Netherlands

**Keywords:** quality of life, resilience, older adults, network analysis, self-management ability, coping, positive appraisal, physical activity

In the published article, there was an error in the labeling of the self-management ability subscales. Specifically, the labels assigned to certain categories of the scale were inadvertently switched, leading to some misinterpretations. The labeling errors occurred in the third, fourth, and fifth subscales. The third subscale was mistakenly labeled as “self-efficacy” when it should have been labeled as “variety.” Similarly, the fourth subscale was incorrectly labeled as “variety” instead of “multifunctionality,” and the fifth subscale was labeled as “multifunctionality” instead of “self-efficacy.”

The main statistical analyses and overall findings remain valid and unaffected. However, the mislabeling of these subscales does yield minor implications for one of our secondary (exploratory) analyses. It turns out that self-efficacy, rather than multifunctionality, plays a significant role in linking self-management ability to quality of life. This is in line with an earlier study of Nieboer et al. (2020), who found that the self-efficacy subscale had the strongest relationship with loneliness. This discovery suggests that interventions aimed at fostering self-efficacy may be the most effective approach in promoting quality of life in later stages of life, rather than multifunctionality of resources.

Several corrections have been made to the text throughout the article.

A correction has been made to the *Abstract*. The corrected *Abstract* is shown below.

Age-related challenges and transitions can have considerable social, psychological, and physical consequences that may lead to significant changes in quality of life (QoL). As such, maintaining high levels of QoL in later life may crucially depend on the ability to demonstrate resilience (i.e., successful adaptation to late-life challenges). The current study set out to explore the interplay between several resilience factors, and how these contribute to the realization and maintenance of (different facets of) QoL. Based on the previous work, we identified behavioral coping, positive appraisal, self-management ability, and physical activity as key resilience factors. Their interplay with (various facets of) QoL, as measured with the WHOQOL-OLD, was established through network analysis. In a sample of community-dwelling older adults (55+; *N* = 1,392), we found that QoL was most strongly (and directly) related to positive appraisal style and self-management ability. Among those, self-efficacy seemed to be crucial. It connected directly to “satisfaction with past, present, and future activities,” a key facet of QoL with strong interconnections to other QoL facets. Our analysis also identified resilience factor(s) with the potential to promote QoL when targeted by training, intervention, or other experimental manipulation. The appropriate set of resilience factors to manipulate may depend on the goal and/or facet of QoL that one aims to improve.

Corrections have also been made to *Results, Exploratory Analyses, How Do Different SMAs Relate to the QoL Facets and Other Resilience Factors?, Paragraphs 1–3*. The corrected paragraphs are shown below.

While earlier analyses suggest that especially SMA is an important factor, it remains unclear what specific self-management abilities are crucially involved. Through exploratory analyses, we aimed to establish whether there are substantial differences in the importance of the six SMA facets included in the SMAS. A third GGM again highlighted that almost all nodes were (in)directly connected to each other and revealed similar associations between the QoL facets (**Figure 5A**; Supplementary Table 4). Edge weights of connections among nodes of the individual facets of the SMAS, as well as the other resilience factors, are reported in **Supplementary Table 5**. Not surprisingly, we observed a particularly strong connection between positive appraisal style (PAS) and the PFM facet of the SMAS, and a relatively weak (and less stable) connection between PAS and MUL (*p* < 0.05, **Figure 5A**; **Supplementary Figures 9**, **10**). Both relationships had similar instrength and outstrength values (*p's* > 0.05; **Figure 5B**), and influenced each other equally. VAR was directly related to physical activity (PHY), with VAR exerting a larger influence on PHY than vice versa (4.7 vs. 2.6%; *p* < 0.05; **Figures 6A**, **C**). This builds on the relationship between SMA and PHY observed in the previous networks and suggests that engaging in physical activity may indirectly enhance QoL, as it helps one to ensure a variety of external resources (e.g., maintaining several friendships) to achieve certain goals in life, but that ensuring such a variety of resources may also strongly (and even to a larger extent than vice versa) promote physical activity. In addition to the previously established connection with PAS, behavioral coping (BC) was also directly related to the VAR (not consistently, 30.6% of the bootstraps set to zero), INI, and SEF facets of the SMAS in this network. Taking all these edges together, the total instrength value of BC was higher compared with the outstrength value (19.0 vs. 15.2%, *p* > 0.05). This is in line with earlier suggestions that boosting (specific) SMAs may not only improve QoL, but also other resilience factors (e.g., BC or PHY) that can indirectly further enhance QoL.

Of all the SMAS facets, SEF had the most direct connections with facets of QoL (i.e., SAB, SOP, INT, PPF; **Figure 5A** and **Supplementary Table 6**) and the largest total outstrength-instrength difference (69.7%−49.8%; *p* < 0.05; **Figures 5B**, **6A**, **C**), even when excluding the relationships with other resilience factors (22.8%−15.7%). However, in the latter situation, the estimation of the difference was relatively unstable, resulting in a large quantile interval that contained zero (*p* > 0.05; **Figures 6B**, **D**). The connection of SEF with the PPF facet (7.7 vs. 6.4%, *p* > 0.05) was of particular interest, since PPF was not directly related to overall SMA in the second GGM. Moreover, this connection appeared to be stronger than the edge between PPF and PAS, although not significantly (*p* > 0.05; **Supplementary Figures 9**, **10**). These exploratory findings suggest that, potentially, when aiming to improve the PPF facet of QoL, one should focus on enhancing one's belief in the competence to achieve certain goals in life, rather than PAS. Due to the considerably high total outstrength value of PPF on other facets of QoL (79.0 vs. 48.4% instrength, *p* < 0.05), this may also be an excellent strategy to indirectly enhance AUT (29.7%), SOP (29.3%), INT (14%), DAD (4.3%), and SAB (1.7%), and thereby QoL as a whole. Several other positive (and some negative) relationships between the individual SMAs and QoL facets were observed as well, although most of them were considerably unstable (see **Figures 5**, **6** and **Supplementary Figures 9**, **10**).

In sum, these exploratory analyses again highlight that the selection of the most appropriate resilience factor to manipulate depends on the quality of life (QoL) facet one aims to promote. Most interestingly, it seems that targeting the self-efficacy facet of the SMAS can potentially have the strongest effect on overall QoL. This facet was strongly related to the past, present, and future (PFF) activities facet of QoL, which in turn exerts a large influence on multiple other QoL facets.

Corrections have also been made to several parts of the *Discussion*, as outlined below.

Corrections have been made to *Discussion, Paragraph 3*. The corrected paragraph is shown below.

The relationship of SMA with QoL was driven by various underlying associations with multiple facets of QoL, including sensory abilities (SAB), social participation (SOP), autonomy (AUT) and intimacy (INT). Moreover, while PAS appeared to influence overall QoL to a lesser extent than SMA, it was strongly connected to specific facets, namely DAD and PPF. Therefore, manipulation of PAS may be an effective pathway for decreasing worries about death and dying and improving satisfaction about achievements in life and at things to look forward to. However, exploratory analyses revealed that specifically targeting the self-efficacy (SEF) facet of the SMAS could also be a promising strategy to improve reports on PPF. Considering the large influence that PPF exerts on other QoL facets, SEF may be an excellent target for interventions or individuals' own efforts to promote overall QoL.

Corrections have been made to *Discussion, Main (and Exploratory) Findings, Paragraphs 2 and 3*. The corrected paragraphs are shown below.

In addition to this main finding, the exploratory analyses on the individual SMAs revealed that SEF was relatively strongly connected to the PPF facet of QoL. This implies that the ability to gain and maintain a belief in one's personal competence, control and self-efficacy in achieving certain goals in life at old age contributes to the extent to which one is satisfied with past, present and future activities. Potentially, individuals with strong self-efficacy beliefs are more likely to undertake the activities and efforts needed to achieve their goals (Steverink et al., 2005), and as a consequence they are more satisfied with achievements in life and things to look forward to. Since PFF has a large outstrength on other facets of QoL, SEF may be a critical target for interventions aiming to improve overall QoL. Indeed, aging involves transitions and changes that introduce new challenges and uncertainties, which can undermine individuals' self-efficacy beliefs (Steverink et al., 2005; Nieboer et al., 2020). This may be due to a sudden physical limitation, or fewer opportunities for social contacts (e.g., interaction with colleagues, physical exercise within a group) and skill development (e.g., learning new things in a working environment), as well as increasing experiences of loss and failure. Hence, building interventions that help individuals to promote SMA, and self-efficacy in particular, thereby reducing potential declines in QoL (and wellbeing), is highly important.

One of the few existing interventions that has been developed for this purpose is the self-management of wellbeing (SMW) intervention, tested in different formats (individual, group and self-help; Schuurmans, 2004; Frieswijk et al., 2006; Kremers et al., 2006; see Goedendorp and Steverink, 2017 for comparison). However, this intervention is high intensive, involving multiple (5–6) session (of 1–2.5 h). To improve the accessibility for older adults, it may be useful to explore possibilities for low intensity interventions that focus on teaching individuals how to successfully adjust their behavior in accordance with internal or external demands and challenges, thereby fostering their belief in the personal competence to achieve life goals in general. Promoting the use of the strategic planning technique of implementation intentions could be useful for this purpose (Gollwitzer, 1999).

A correction has been made to *Discussion, Main (and Exploratory) Findings, Paragraph 6*. The corrected paragraph is shown below.

Our findings provide relatively strong support for our hypothesis that physical activity has a positive effect on QoL (e.g., Windle et al., 2010), either directly or by promoting other resilience factors (e.g., Ávila et al., 2018). That is, PHY was positively associated and linked to SMA, and therefore indirectly to QoL, but the relative importance as a predictor of SMA was low in comparison to its instrength (from SMA). This implies that SMA mediated the relationship between PHY and QoL, but that the contribution of SMA on PHY was larger. This is in line with the idea that strengthening resilience can improve the adherence to exercise behaviors (Resnick and Inguito, 2011). Interestingly, our exploratory analyses revealed that the relationship between PHY and SMA was driven by the variety subscale specifically. This implies that having a variety of external resources to achieve a certain life goal, such as engaging in multiple hobbies or in versatile volunteer activities, or having a diverse network of friends or engaging in various different group activities, can promote regular participation in physical activities. Indeed, having multiple hobbies inherently increases the likelihood that at least one of those involves physical exercise. In addition, one's social connections can play a vital role in promoting health-oriented behaviors, and having a wide circle of friends may encourage physical activity by providing support and companionship (Smith et al., 2017; Schlenk et al., 2021). In turn, physical activity, particularly group exercise classes and team sports, is likely to foster additional social interactions and personal growth that may contribute to one's ability to ensure a variety of external resources. Thus, our results suggest that engaging in physical activities can positively shape areas of an individual's life beyond the physical health, and that enhancing one's ability to acquire and maintain a variety of resources may greatly enhance overall QoL, as well as engagement in physical activity specifically. The fact that PHY had no direct associations with QoL remains surprising. A possible explanation may be that the current measure of PHY was too general and did not pick up decisive differences among individuals. This has been shown to be a general pitfall of self-report measures of physical activity (Prince et al., 2008), which emphasizes that cautious interpretation is warranted.

A correction has also been made to section *Concluding remarks*. The corrected section is shown below.

Summarizing, this study contributes to our understanding of the interplay between factors that underpin resilience in later life. We have provided evidence to suggest that SMA and, to a lesser extent, PAS are most crucially involved in the realization and maintenance of high levels of QoL, and building interventions targeting these factors therefore seems most promising when trying to improve QoL. Teaching older adults how they can successfully adjust their behavior to achieve specific life goals, thereby promoting self-efficacy beliefs, may be an excellent starting point for interventions. However, the appropriate set of resilience factors to manipulate may ultimately depend on the facet of QoL that one intends to improve. These findings can aid future studies in determining specific strategies that can help older adults to gain control of their own lives, enabling them to maintain the functional ability and competence that is vital for wellbeing and QoL at old age.

In the published article, there was also an error in Table 1 as published. Some of the labels of the subscale of the self-management ability scale were switched. The corrected Table 1 and its caption appear below.

**Table 1:** Descriptive statistics of the QoL (facets) and the resilience factors for all participants.

**Construct (abbreviation; possible range)**	***M* (SD)**	**Observed range**
Quality of life (QoL; 24–100)	94.6 (9.52)	51–120
Sensory abilities (SAB; 4–20)	17.6 (2.61)	6–20
Autonomy (AUT; 4–20)	15.9 (2.00)	7–20
Past, present, and future activities (PPF; 4–20)	15.9 (2.07)	6–20
Social participation (SOP; 4–20)	15.7 (2.61)	5–20
Death and dying (DAD; 4–20)	14.7 (2.89)	4–20
Intimacy (INT; 4–20)	14.9 (2.92)	4–20
Behavioral coping (BC; 8–32)	21.5 (3.71)	9–32
Positive appraisal style (PAS)^a^	0.03 (0.60)	−1.78 to 1.72
Self-management ability (SMA; 0–100)	69.2 (11.6)	28.9–98.9
Taking initiative (INI; 0–100)	69.7 (16.7)	20–100
Investment behavior (INV; 0–100)	77.0 (15.6)	20–100
Variety (VAR; 0–100)	58.6 (16.14)	6.67–100
Multifunctionality (MUL; 0–100)	60.8 (16.5)	6.67–100
Self-efficacy (SEF; 0–100)	83.2 (14.9)	25–100
Positive frame of mind (PFM; 0–100)	66.0 (17.3)	0–100
Physical activity (PHY)^b^	3,641.6 (2,109.6)	0–9,180
Stringency Index (SI; 0–100)	65.9 (8.4)	56.48–82.41

^*a*^z-score.

^*b*^Composite score: physical activity duration weighted by MET.

Additionally, there were also errors in Figures 5, 6 as published. Some of the labels of the subscale of the self-management ability scale are switched. The corrected figures and their captions appear below.

**Figure 5:**
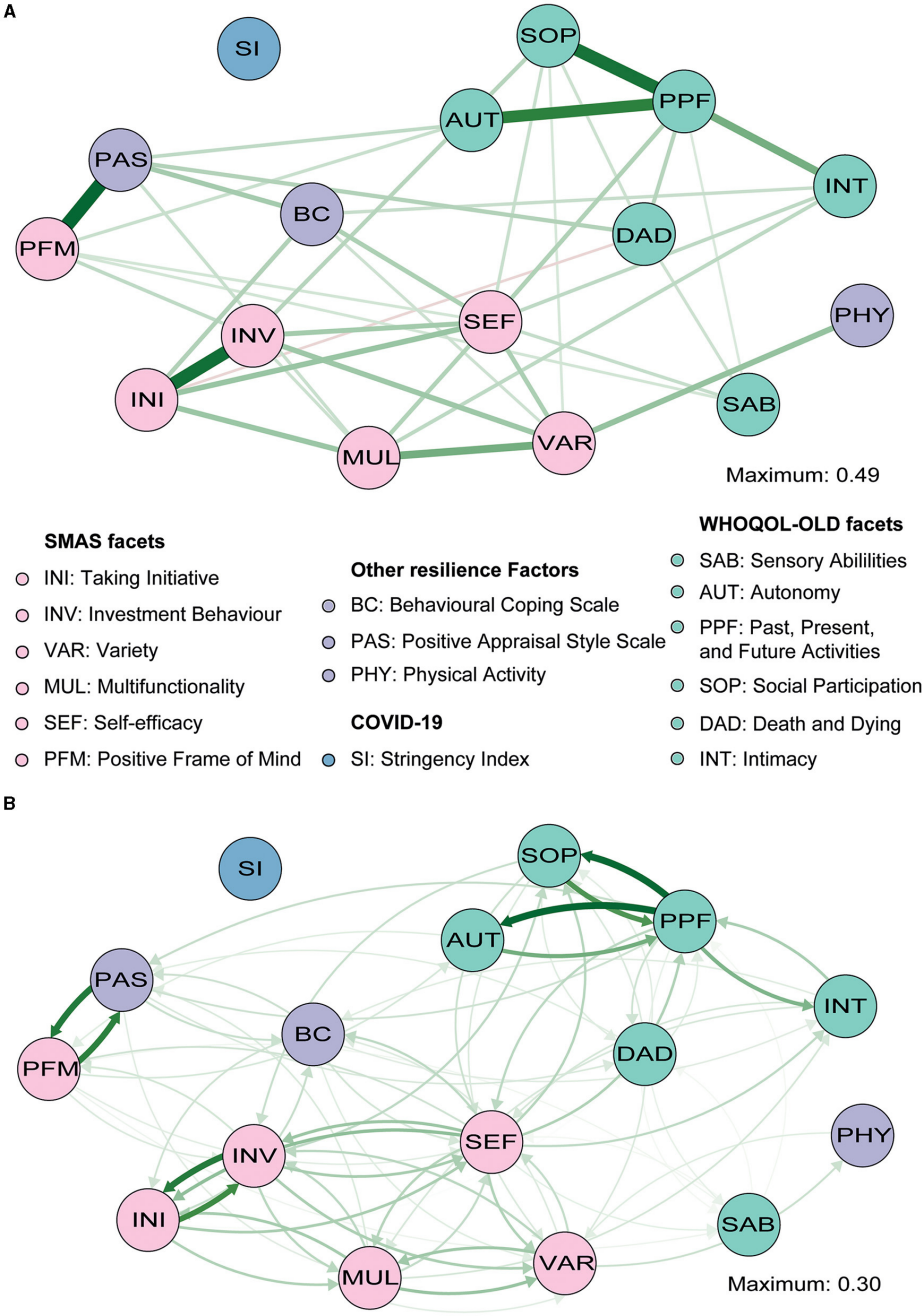
Gaussian graphical model [GGM; **(A)**] and directed relative importance network **(B)** of individual facets of QoL (green), the facets of the SMAS (pink) and other resilience factors (purple), and the stringency index (blue). The maximum value represents the highest edge weights included in the network.

**Figure 6:**
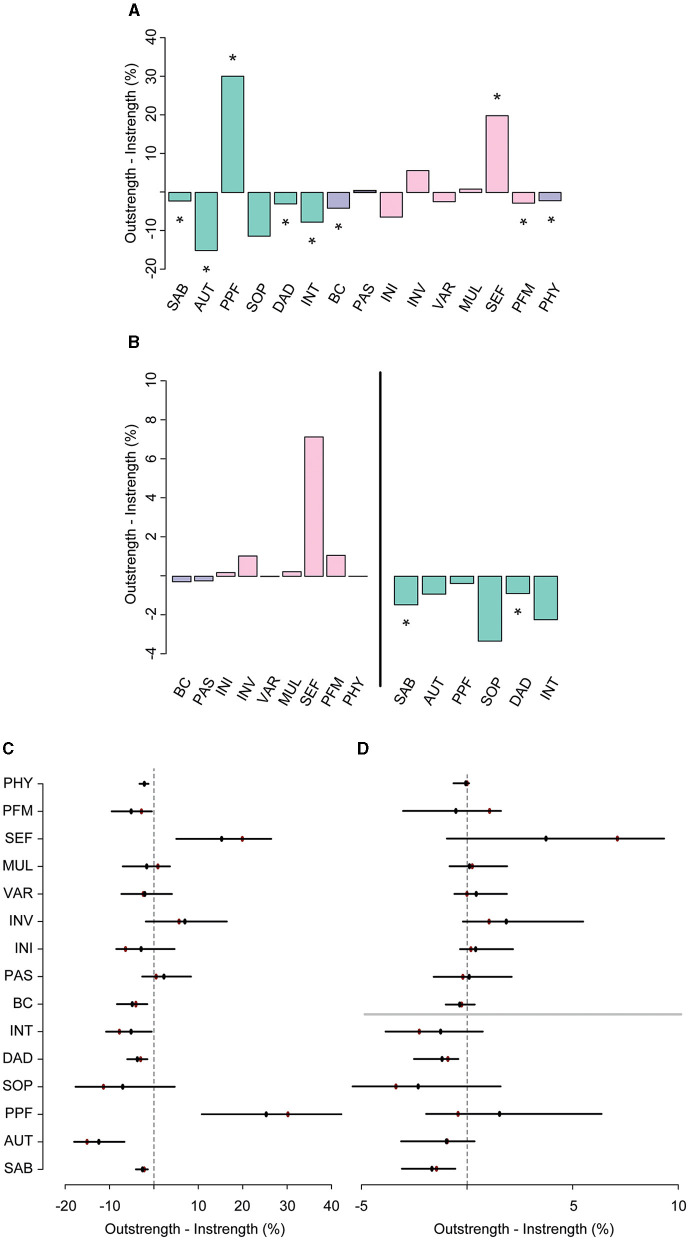
The difference between overall outstrength and instrength of the nodes in the third network **(A)**, and the difference in outstrength and instrength of the relationships between the resilience factors and QoL facets only [**(B)**, left], and the relationships between the QoL facets and the resilience factors [**(B)**, right]. Colors correspond to the nodes in the network in Figure 5. In plots **(C, D)**, the bootstrapped mean is depicted in black and the sample mean in red. ^*^p < 0.05, nodes with quantile intervals containing zero are deemed to have an insignificant instrength and outstrength difference.

Lastly, in the published article, there were errors in the Supplementary material.

There were errors in Supplementary Tables 5, 6. Some of the labels of the subscale of the self-management ability scale were switched. The corrected tables appear below.

**Supplementary Table 5:** Edge weights (r) of the connections among all resilience factors in the third, exploratory GGM.

	**BC**	**PAS**	**INI**	**INV**	**VAR**	**MUL**	**SEF**	**PFM**	**PHY**
**BC**		0.15	0.12	0	0.07	0	0.14	0	0
**PAS**			0	0	0	0.08	0	0.49	0
**INI**				0.47	0	0.17	0.17	0	0
**INV**					0.16	0.08	0.15	0.10	0
**VAR**						0.26	0.14	0	0.18
**MUL**							0.12	0	0
**SEF**								0.07	0
**PFM**									0

**Supplementary Table 6:** Edge weights (r) of the connections between the resilience factors and QoL facets in the third, exploratory GGM.

	**SAB**	**AUT**	**PPF**	**SOP**	**DAD**	**INT**
**BC**	0	0	0	0	0	0.09
**PAS**	0	0	0.1	0	0.12	0
**INI**	0	0	0	0	−0.06	0
**INV**	0	0	0	0.12	0	0
**VAR**	0	0	0	0.06	0	0
**MUL**	0	0	0	0	0	0.10
**SEF**	0.09	0	0.13	0.08	0	0.10
**PFM**	0.07	0.08	0	0	0	0
**PHY**	0	0	0	0	0	0

Finally, there were errors in Supplementary Figures 9, 10. Some of the labels of the subscale of the self-management ability scale were switched. The corrected figures and legends appear below.

**Supplementary Figure 9:**
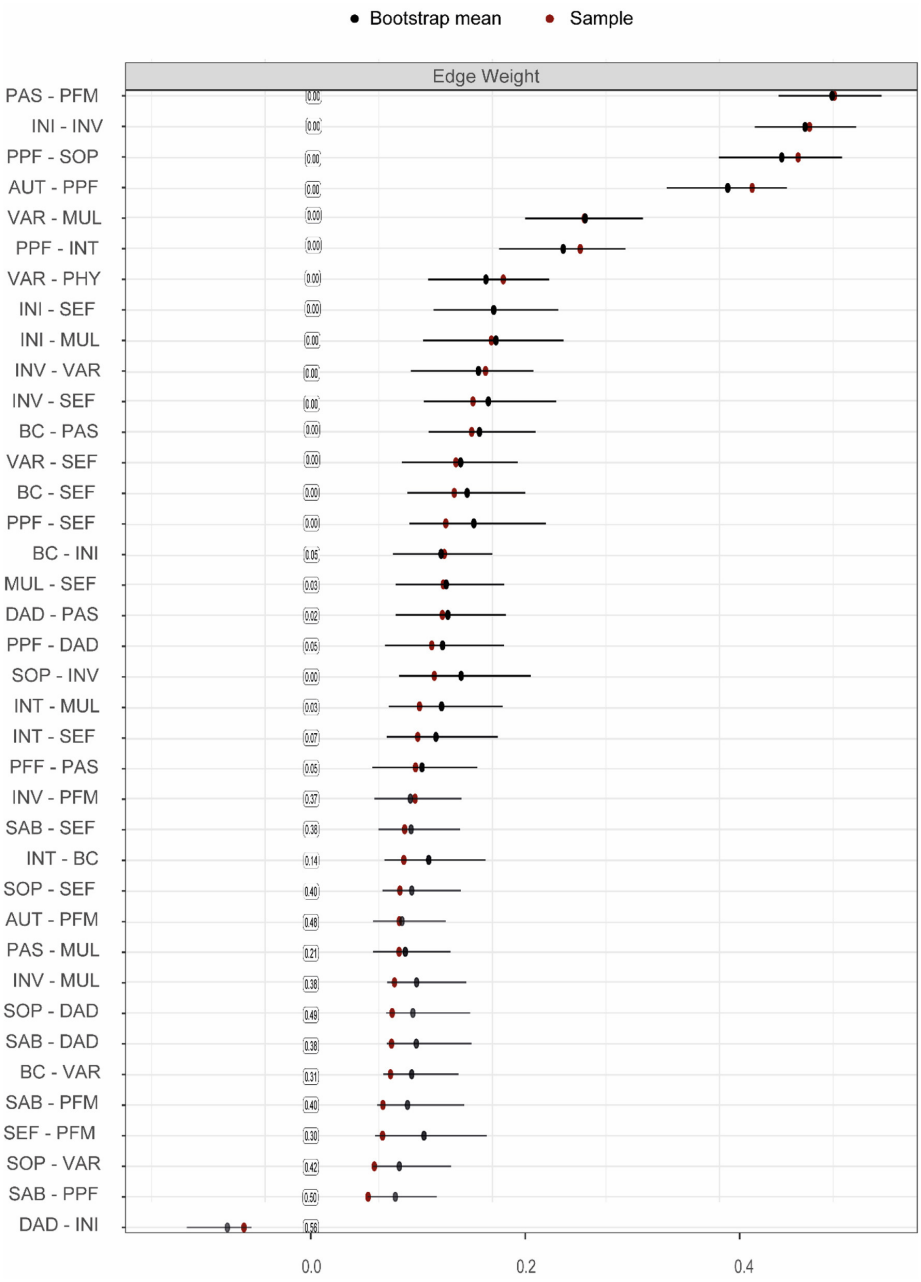
Bootstrap and sample means, including quantile intervals (only for the times the parameter was not set to zero) around the bootstrap mean for edge weights of the third, exploratory GGM. The values in the boxes represent the probability of how often the parameter was estimated set to zero. Note that this figure only includes the edges that are included in the network, to improve readability.

**Supplementary Figure 10:**
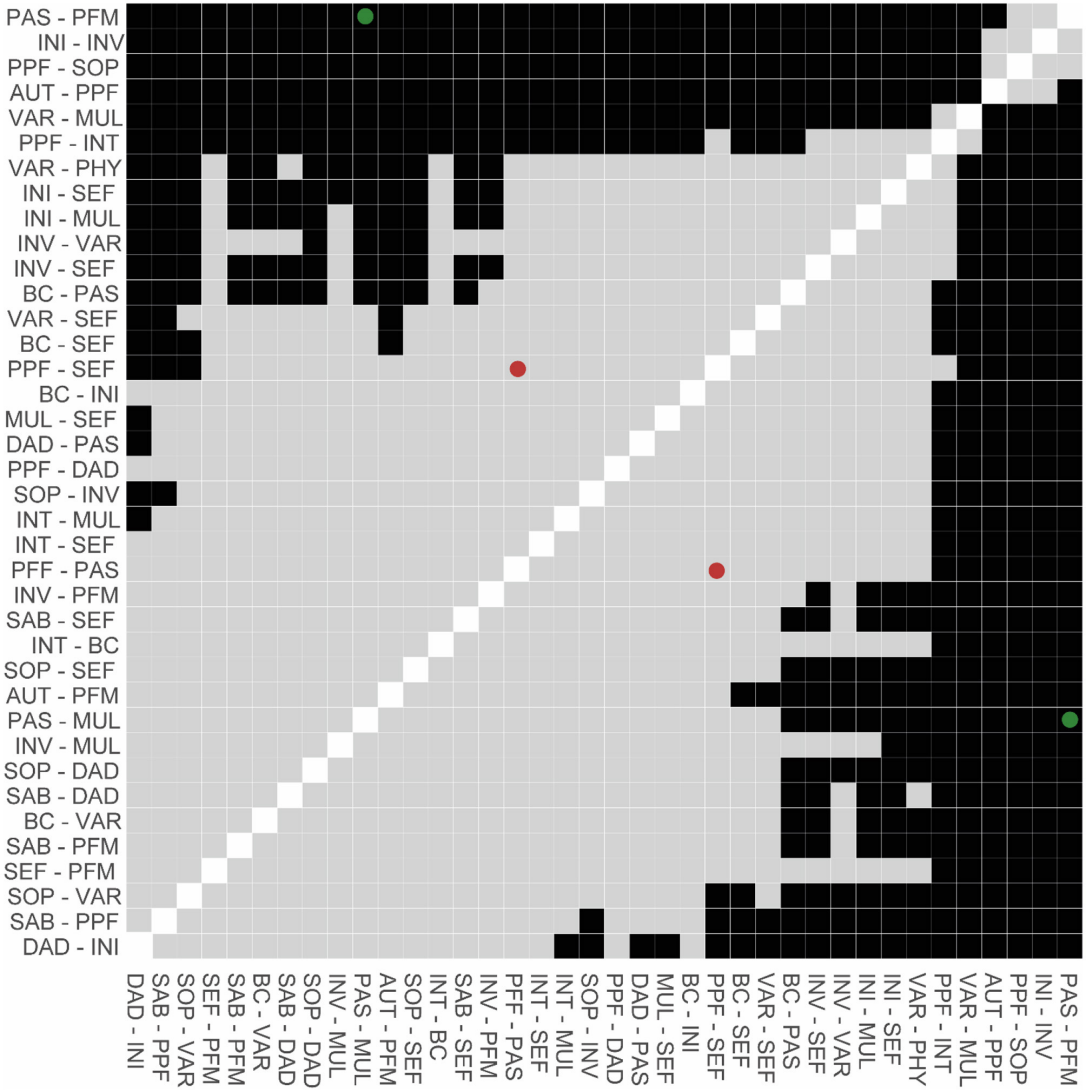
Difference plot of edge weights of the third, exploratory GGM. Black squares depict significant differences in edge weights (*p* < 0.05), whereas gray squares illustrate non-significant comparisons (*p* > 0.05). Dots (green = significant, red = insignificant) highlight the most relevant comparisons that are mentioned in text.

The authors apologize for these errors and state that they do not change the scientific conclusions of the article in any way. The original article and its Supplementary material have been updated.
