# Supplementary material for: The Interplay Between Quality of Life and Resilience Factors in Later Life: A Network Analysis
Source: Front Psychol. 2021 Nov 15;12:752564. doi: 10.3389/fpsyg.2021.752564 (PMC8634099; doi:10.3389/fpsyg.2021.752564)
Supplement: Supplementary file 1 [file Data_Sheet_1.PDF]

Supplementary Material

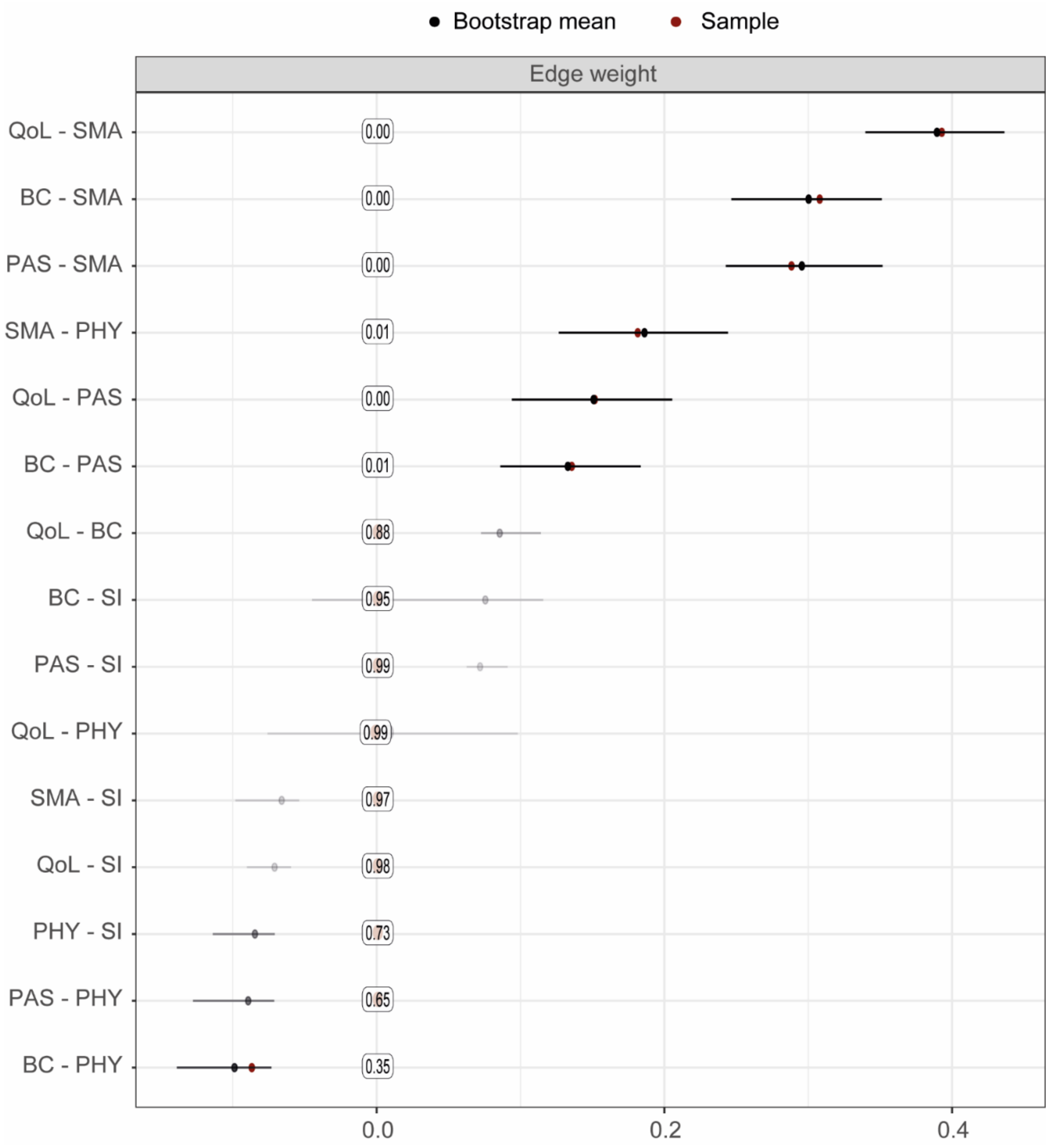

Supplementary Figure 1. Bootstrap and sample means, including QIs (only for the times the parameter was not set to zero) around the bootstrap mean for edge weights of the primary GGM. The values in the boxes represent the probability of how often the parameter was estimated set to zero.

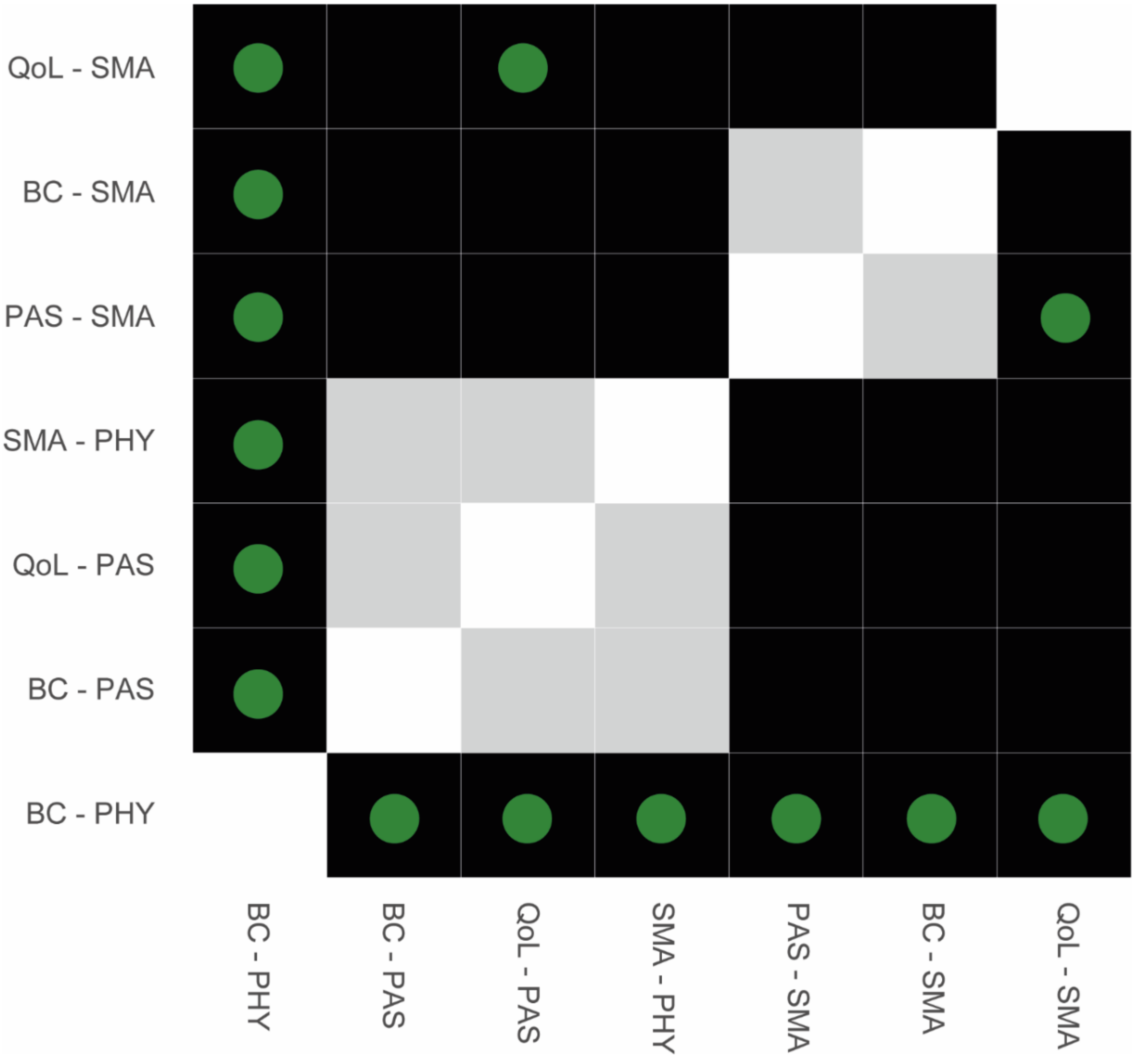

Supplementary Figure 2. Difference plot of edge weights of the primary GGM. Black squares depict significant different in edge weights ($p < .05$), whereas grey squares illustrate non-significant comparisons ($p > .05$). Comparisons were deemed significant if zero was not in the QIs that was constructed on the difference of the two edge weights. Green dots highlight the most relevant comparisons that are mentioned in the main text.

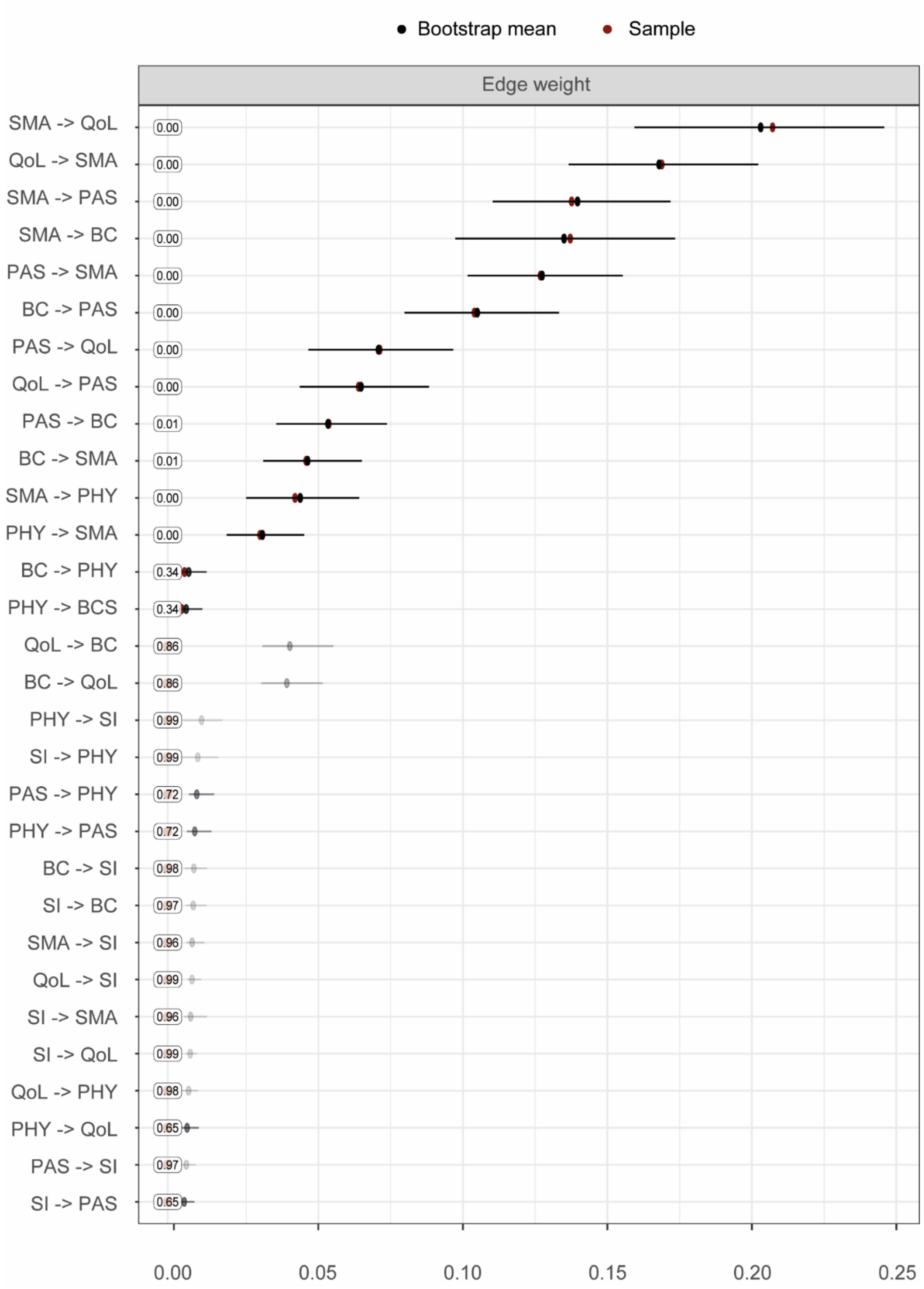
Bootstrap mean
Sample
Edge weight
SMA -> QoL
QoL -> SMA
SMA -> PAS
SMA -> BC
PAS -> SMA
BC -> PAS
PAS -> QoL
QoL -> PAS
PAS -> BC
BC -> SMA
SMA -> PHY
PHY -> SMA
BC -> PHY
PHY -> BCS
QoL -> BC
BC -> QoL
PHY -> SI
SI -> PHY
PAS -> PHY
PHY -> PAS
BC -> SI
SI -> BC
SMA -> SI
QoL -> SI
SI -> SMA
SI -> QoL
QoL -> PHY
PHY -> QoL
PAS -> SI
SI -> PAS
0.00
0.00
0.00
0.00
0.00
0.00
0.00
0.00
0.01
0.01
0.00
0.00
0.34
0.34
0.86
0.86
0.99
0.99
0.72
0.72
0.98
0.97
0.96
0.99
0.96
0.99
0.98
0.65
0.97
0.65
0.00
0.05
0.10
0.15
0.20
0.25

Supplementary Figure 3. Bootstrap and sample means, including quantile intervals (only for the times the parameter was not set to zero) around the bootstrap mean for edge weights of the primary directed relative importance networks.

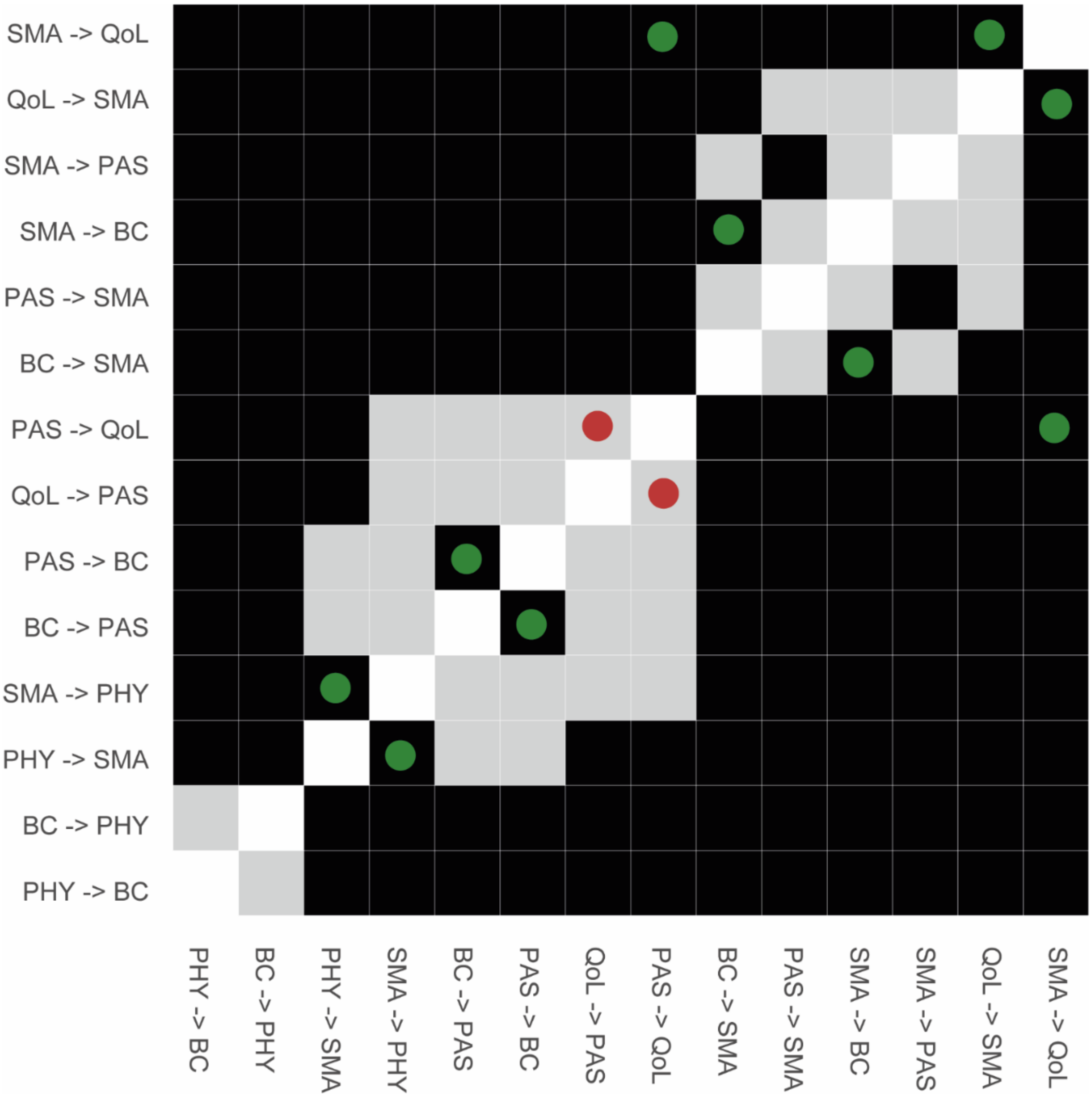

Supplementary Figure 4. Difference plot of the directed edge weights of the primary relative importance network. Black squares depict significant different in the edges ($p < .05$), whereas grey squares illustrate non-significant comparisons ($p > .05$). Dots (green = significant, red = insignificant) highlight the most relevant comparisons.

Supplementary Table 1. Edge weights (r) of the connections among resilience factors in the second GGM.

	BC	PAS	SMA	PHY
**BC**		0.12	0.28	-0.09
**PAS**			0.29	0
**SMA**				0.18

Supplementary Table 2. Edge weights (r) of the connections among QoL facets in the second GGM.

	SAB	AUT	PPF	SOP	DAD	INT
**SAB**		0	0.06	0	0.07	0
**AUT**			0.40	0	0	0
**PPF**				0.48	0.14	0.28
**SOP**					0	0
**DAD**						0

Supplementary Table 3. Edge weights (r) of the connections between QoL facets and the resilience factors in the second GGM.

	SAB	AUT	PPF	SOP	DAD	INT
**BC**	0	0	0	0	0	0.11
**PAS**	0	0	0.11	0	0.13	0
**SMA**	0.12	0.10	0	0.24	0	0.11
**PHY**	0	0	0	0	0	0

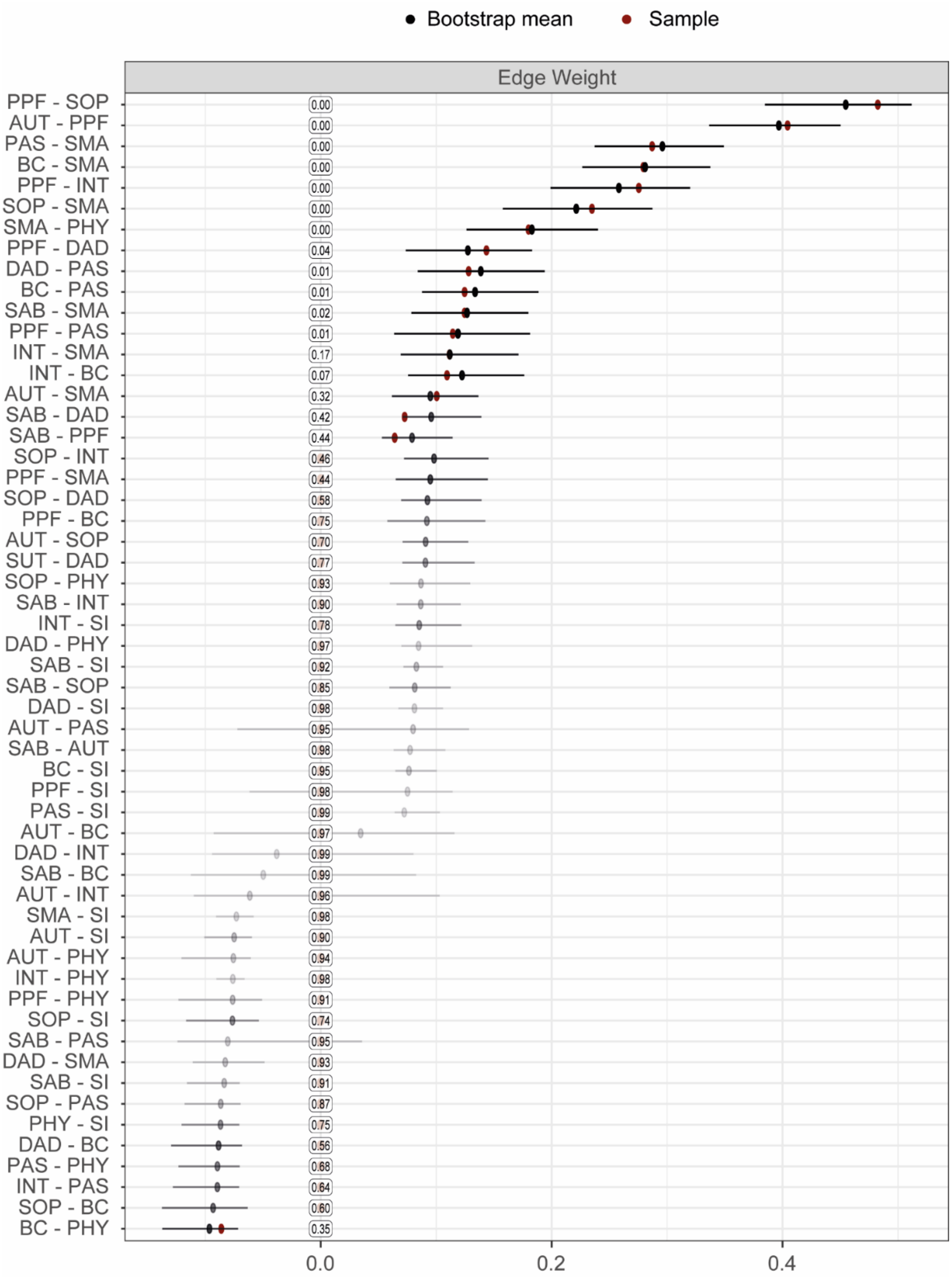

Supplementary Figure 5.. Bootstrap and sample means, including quantile intervals (only for the times the parameter was not set to zero) around the bootstrap mean for edge weights of the second

GGM. The values in the boxes represent the probability of how often the parameter was estimated set to zero.

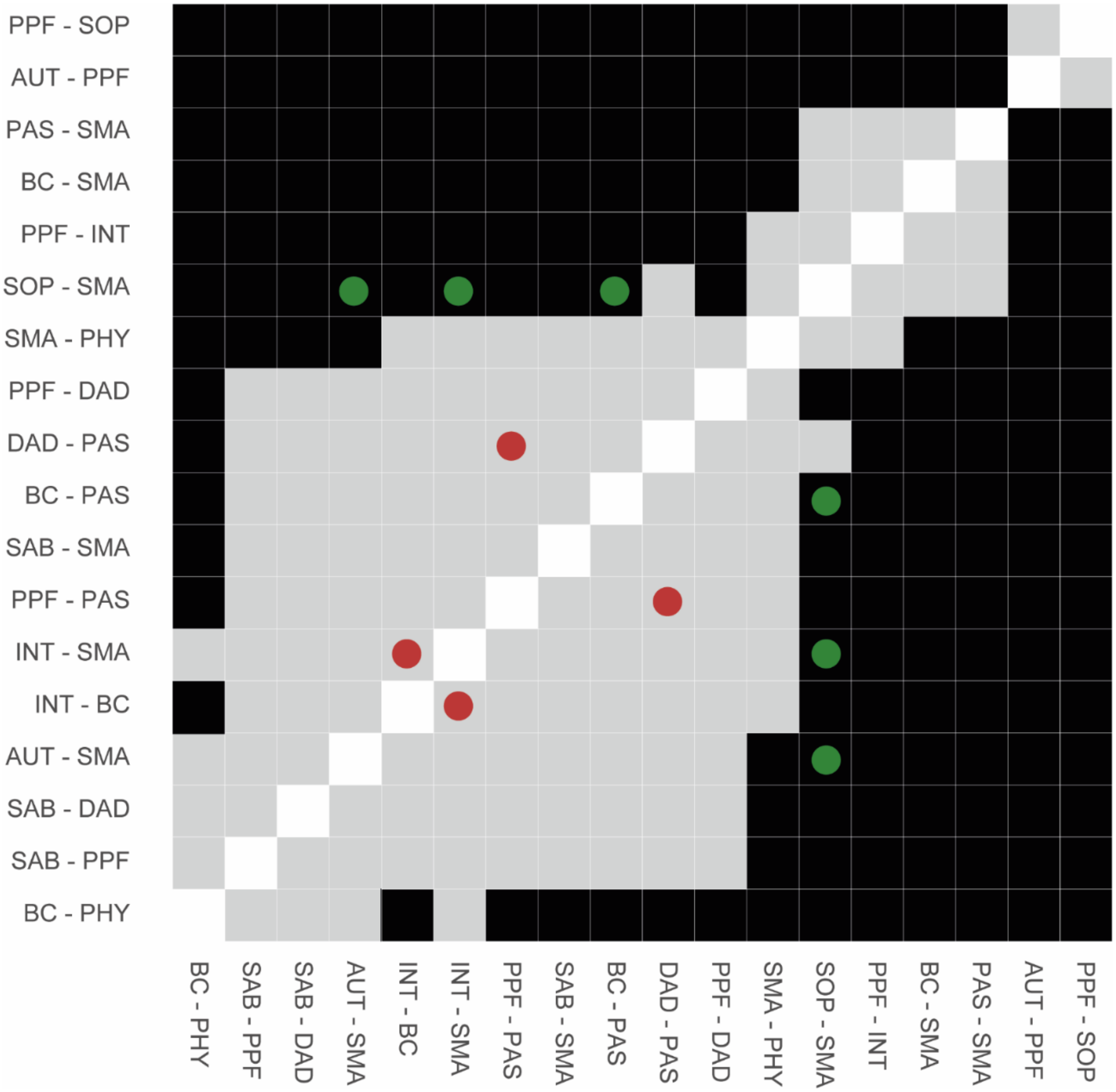

Supplementary Figure 6. Difference plot of edge weights of the second GGM. Black squares depict significant different in edge weights (p < .05), whereas grey squares illustrate non-significant comparisons (p > .05). Dots (green = significant, red = insignificant) highlight the most relevant comparisons that are mentioned in the main text.

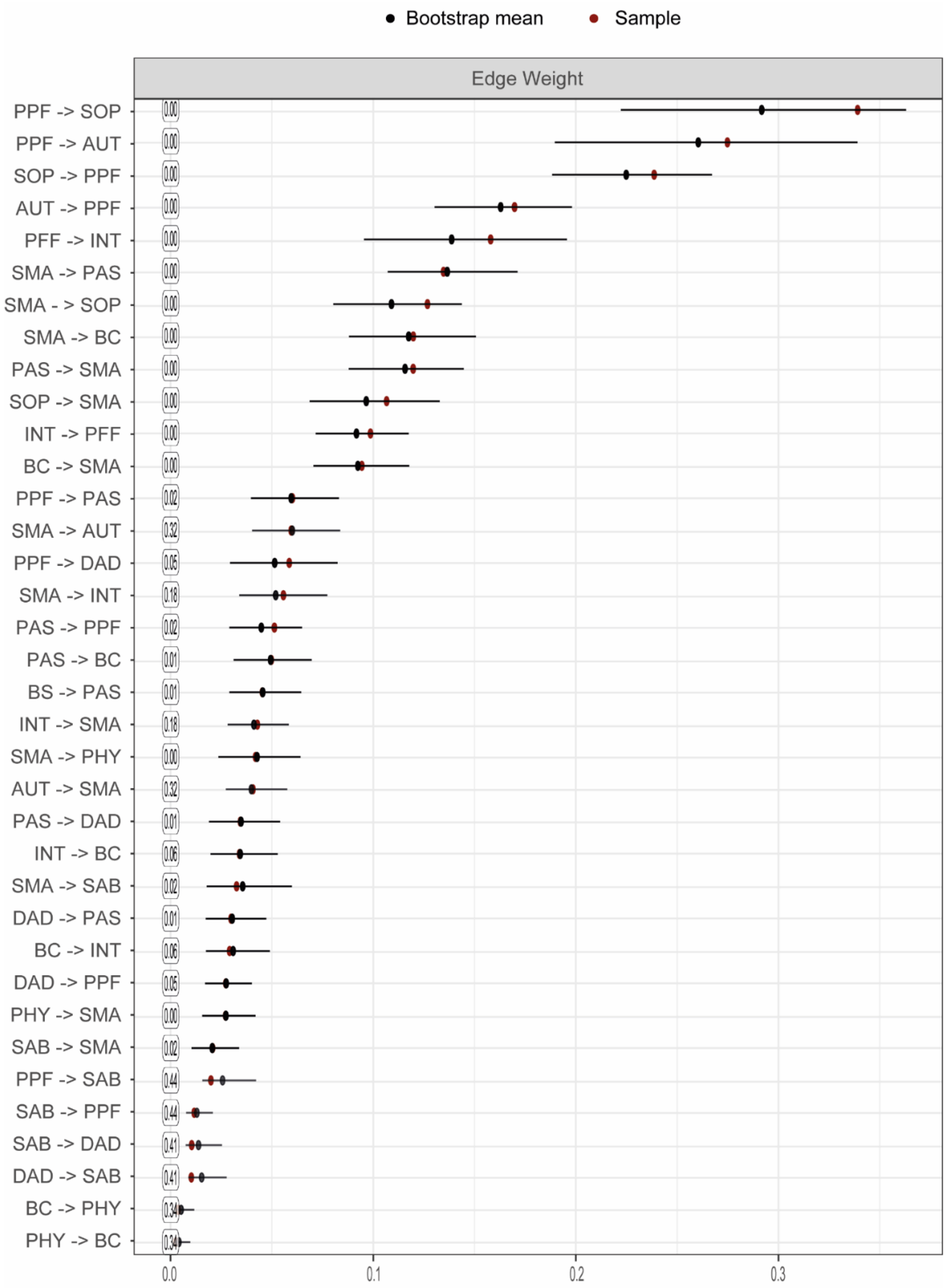

Supplementary Figure 7. Bootstrap and sample means, including quantile intervals (only for the times the parameter was not set to zero) around the bootstrap mean for edge weights of the second

directed relative importance networks. Note that this figure only includes the directed edges that are included in the network, to improve readability.

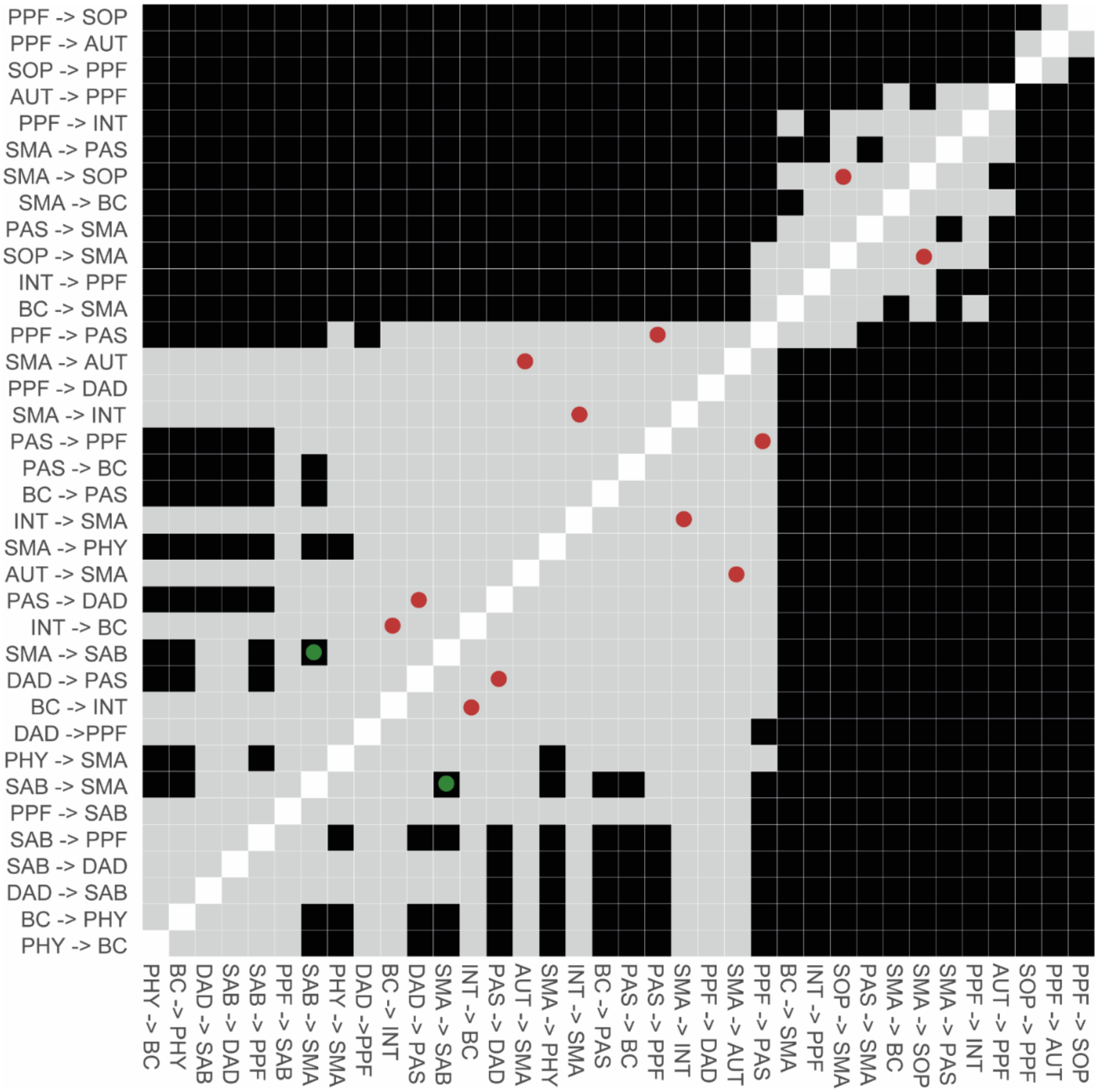

Supplementary Figure 8. Difference plot of the directed edge weights of the second relative importance network. Black squares depict significant different in the edges ($p < .05$), whereas grey squares illustrate non-significant comparisons ($p > .05$). Dots (green = significant, red = insignificant) highlight the most relevant comparisons.

Supplementary Table 4. Edge weights (r) of the connections among QoL facets in the third, exploratory GGM.

	SAB	AUT	PPF	SOP	DAD	INT
**SAB**		0	0.05	0	0.08	0
**AUT**			0.41	0	0	0
**PPF**				0.45	0.12	0.25
**SOP**					0.08	0
**DAD**						0

Supplementary Table 5. Edge weights (r) of the connections among all resilience factors in the third, exploratory GGM.

	BC	PAS	INI	INV	SEF	VAR	MUL	PFM	PHY
**BC**		0.15	0.12	0	0.07	0	0.14	0	0
**PAS**			0	0	0	0.08	0	0.49	0
**INI**				0.47	0	0.17	0.17	0	0
**INV**					0.16	0.08	0.15	0.10	0
**SEF**						0.26	0.14	0	0.18
**VAR**							0.12	0	0
**MUL**								0.07	0
**PFM**									0

Supplementary Table 6. Edge weights (r) of the connections between the resilience factors and QoL facets in the third, exploratory GGM.

	SAB	AUT	PPF	SOP	DAD	INT
**BC**	0	0	0	0	0	0.09
**PAS**	0	0	0.1	0	0.12	0
**INI**	0	0	0	0	-0.06	0
**INV**	0	0	0	0.12	0	0
**SEF**	0	0	0	0.06	0	0
**VAR**	0	0	0	0	0	0.10
**MUL**	0.09	0	0.13	0.08	0	0.10
**PFM**	0.07	0.08	0	0	0	0
**PHY**	0	0	0	0	0	0

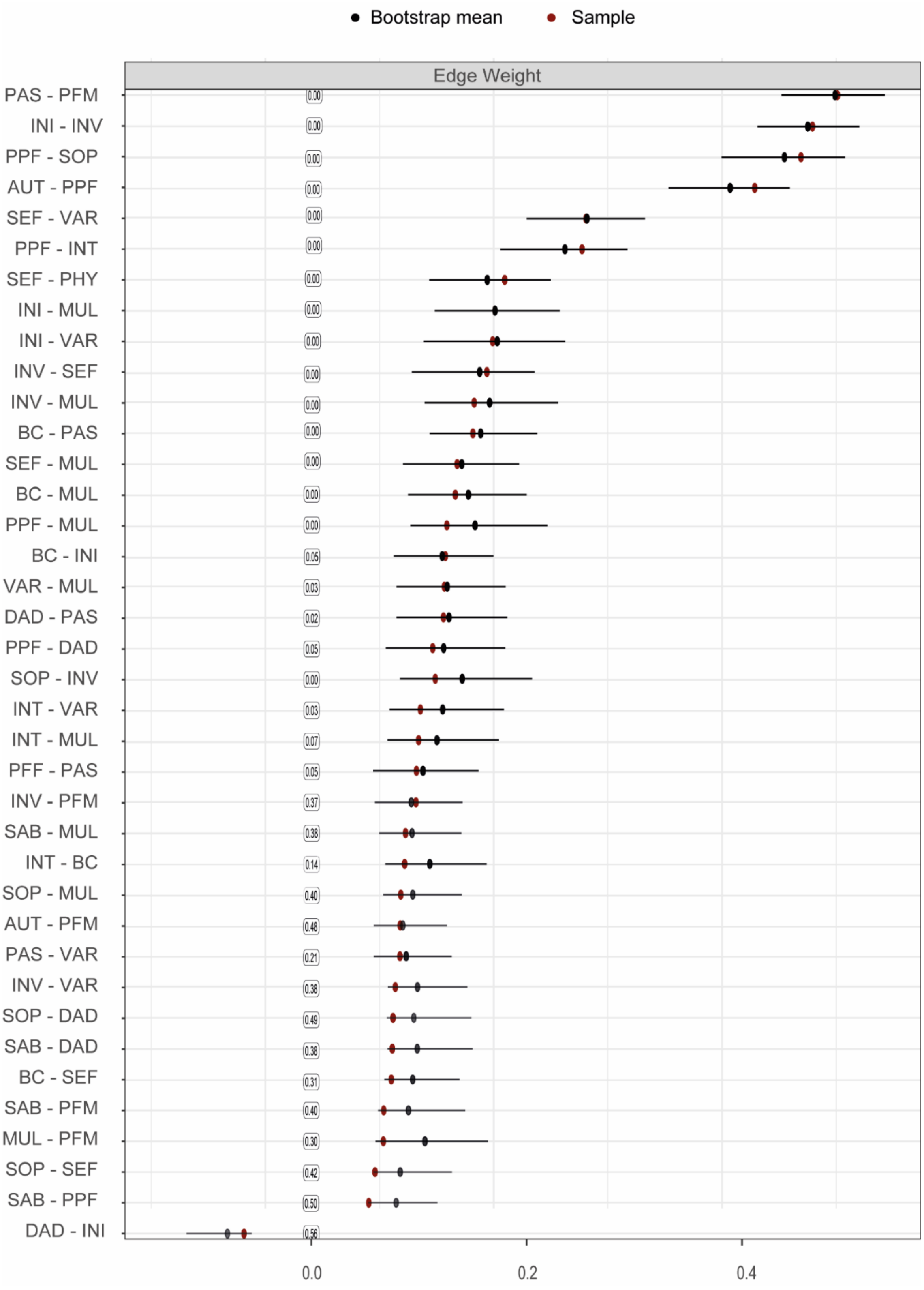

Supplementary Figure 9. Bootstrap and sample means, including quantile intervals (only for the times the parameter was not set to zero) around the bootstrap mean for edge weights of the third, exploratory GGM. The values in the boxes represent the probability of how often the parameter was

estimated set to zero. Note that this figure only includes the edges that are included in the network, to improve readability.

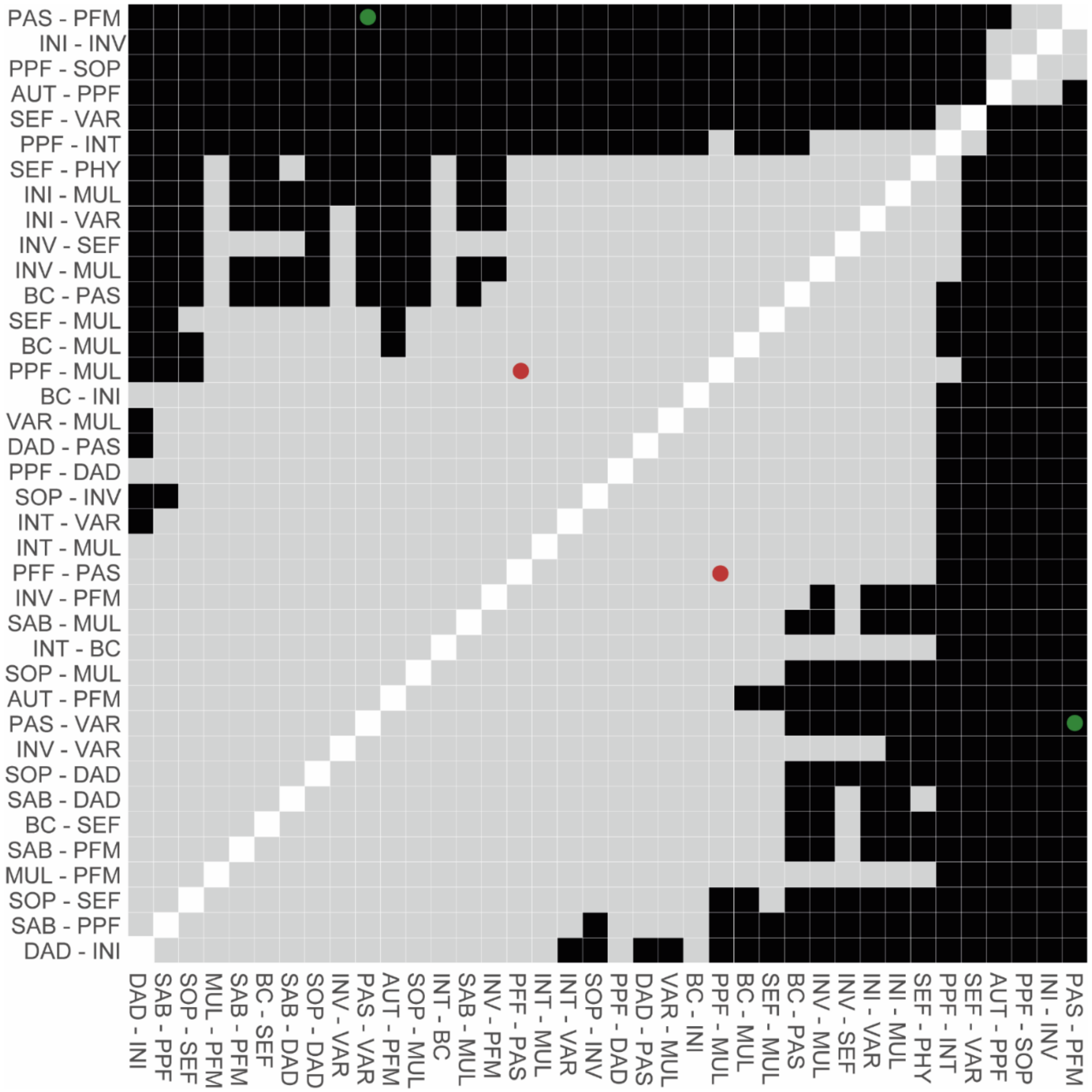

Supplementary Figure 10. Difference plot of edge weights of the third, exploratory GGM. Black squares depict significant differences in edge weights ($p < .05$), whereas grey squares illustrate non-significant comparisons ($p > .05$). Dots (green = significant, red = insignificant) highlight the most relevant comparisons that are mentioned in text.
